# Sensing with Femtosecond Laser Filamentation

**DOI:** 10.3390/s22187076

**Published:** 2022-09-19

**Authors:** Pengfei Qi, Wenqi Qian, Lanjun Guo, Jiayun Xue, Nan Zhang, Yuezheng Wang, Zhi Zhang, Zeliang Zhang, Lie Lin, Changlin Sun, Liguo Zhu, Weiwei Liu

**Affiliations:** 1 Institute of Modern Optics, Eye Institute, Nankai University, Tianjin 300350, China; 2 Tianjin Key Laboratory of Micro-Scale Optical Information Science and Technology, Tianjin 300350, China; 3 Tianjin Key Laboratory of Optoelectronic Sensor and Sensing Network Technology, Tianjin 300350, China; 4 National Key Laboratory of Shock Wave and Detonation Physics, Institute of Fluid Physics, China Academy of Engineering Physics, Mianyang 621900, China

**Keywords:** femtosecond laser, filamentation, remote sensing

## Abstract

Femtosecond laser filamentation is a unique nonlinear optical phenomenon when high-power ultrafast laser propagation in all transparent optical media. During filamentation in the atmosphere, the ultrastrong field of 10^13^–10^14^ W/cm^2^ with a large distance ranging from meter to kilometers can effectively ionize, break, and excite the molecules and fragments, resulting in characteristic fingerprint emissions, which provide a great opportunity for investigating strong-field molecules interaction in complicated environments, especially remote sensing. Additionally, the ultrastrong intensity inside the filament can damage almost all the detectors and ignite various intricate higher order nonlinear optical effects. These extreme physical conditions and complicated phenomena make the sensing and controlling of filamentation challenging. This paper mainly focuses on recent research advances in sensing with femtosecond laser filamentation, including fundamental physics, sensing and manipulating methods, typical filament-based sensing techniques and application scenarios, opportunities, and challenges toward the filament-based remote sensing under different complicated conditions.

## 1. Introduction

The filamentation can be dated back to the long damage chains with a diameter of a few microns by focusing “long” laser pulses (ns down to ps) in solids, where various promising nonlinear phenomena were masked by the laser-induced breakdown that the medium gets totally ionized through collision processes [1]. The femtosecond laser featured with ultrastrong peak power and ultrashort pulse duration avoids the conventional optical breakdown and makes a huge difference [2,3,4,5,6,7,8], reigniting intensive interest in exploring such phenomenon. During the femtosecond laser pulses propagate in transparent media including gases, liquids, and solids, the beam can propagates over a long distance without diffraction along with a self-generated plasma channel [1,9], as a result of the dynamic counteraction of the optical Kerr self-focusing effect and the plasma defocusing effect [10]. Due to the different free electron generation mechanisms, the obvious difference is the plasma density and length of the filament for gases and condensed matter materials. The unique femtosecond laser filamentation encompasses abundant nonlinear optical phenomena, including group velocity dispersion (GVD), self-focusing, self-phase modulation (SPM), self-steepening, multi-photon and tunneling ionization (MPI/TI), multi-photon absorption (MPA), higher harmonics, Cherenkov radiation, stimulated amplification, spatio-temporal coupling, stimulated Raman effect, molecular alignment, and multifilament competition, providing a versatile platform to explore the promising applications of atmospheric remote sensing, lightning control, ultra-broadband light generation, strong terahertz wave emission, and laser-induced water condensation [1,9,11].

The femtosecond laser filamentation can give rise to a high nearly constant laser intensity of about 10^13^–10^14^ W/cm^2^ in a large distance ranging from meters to kilometers in the atmosphere [12,13]. The ultrastrong laser intensity is high enough to induce remote ionization and fragmentation of molecules, giving rise to characteristic fingerprint fluorescence emissions. This opens up the possibility of investigating strong-field molecule interaction at a remote place in a variety of complicated environments, especially the remote sensing for identifying parent molecules [14]. Therefore, toward the remote sensing in different complicated conditions, scientists devote tremendous efforts to the sensing with femtosecond laser filamentation from fundamental physics to applications in the past two decades. Specifically, the underlying physics of femtosecond laser filamentation have been firstly explored and are now basically understood. However, the ultrastrong intensity inside the filament can damage almost all the detectors and ignite various intricate higher order nonlinear optical effects. In terms of temporal and spatial scale, the filaments possess a diameter of hundreds of micrometers but a length ranging from meters to kilometers, as well as the ultrafast laser pulses that constantly generate new spectral components during propagation, leading to the complex self-steepening and self-compressing on the duration and shape of the pulses. Consequently, these extreme conditions and complicated phenomena make the sensing and controlling of filamentation challenging. Fortunately, the abundant energy conversion effects among light, acoustic, and thermal signals during filamentation open a door to explore and diagnose the filamentation physics. So far, various methods have been developed to diagnose and manipulate the filamentation, paving the way to all kinds of promising applications that are based on powerful filamentation. As a typical case, versatile filament-based sensing technologies have been also developed and applied to different scenarios.

**Figure 1:**
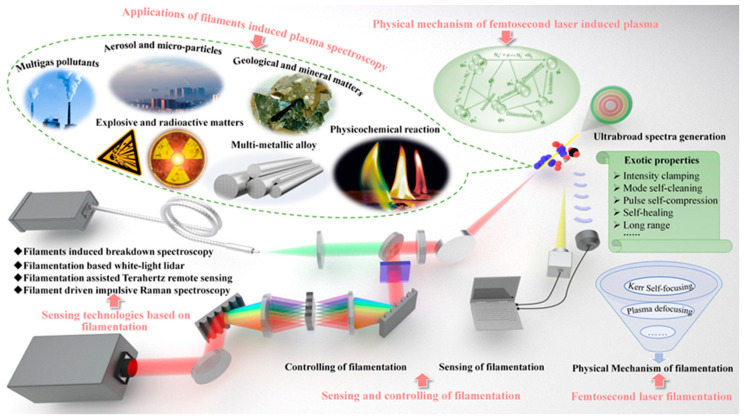
Overview of the recent progress in sensing with femtosecond laser filamentation.

The main scope of this review is aiming to present the recent advances in sensing with femtosecond laser filamentation from basic physics and manipulated methods of filamentation to the representative filament-based sensing techniques and application scenarios and discuss the opportunities and challenges toward the filament-based remote sensing under different complicated conditions. As illustrated in Figure 1, the content is organized as follows. In Section 2, we firstly focus on the unique nonlinear optical phenomenon—femtosecond laser filamentation, including the physical mechanism, exotic properties, and the ultrabroad spectra generation that is closely related to our theme of sensing. Section 3 provides an overview of the sensing techniques and control methods of filamentation, which lay the foundation for the filament-based sensing techniques. Then the versatile sensing technologies that are based on filamentation including filament-induced breakdown spectroscopy, filamentation-based white-light LIDAR (light detection and ranging), filamentation-assisted terahertz remote sensing, and filament-driven impulsive Raman spectroscopy are discussed in Section 4. Concentrating on the most common filament-induced breakdown spectroscopy, the physical mechanism of femtosecond laser-induced breakdown in transparent optical media (gases, liquids, and solids) and the various sensing applications are summarized in Section 5 and Section 6, respectively. Finally, the challenges and perspectives in the filament-based remote sensing are discussed.

## 2. Femtosecond Laser Filamentation

**Figure 2:**
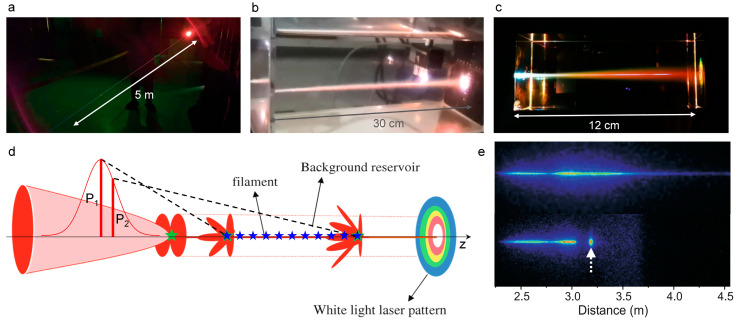
Physical mechanism of femtosecond laser filamentation. (**a**–**c**) Femtosecond laser filamentation in air (**a**), liquid (**b**), and solid (**c**). (**d**) Schematic diagram of the evolution of a femtosecond laser pulse propagating in an optical medium that is based on the moving focus model. The central slice of the pulse self-focuses to a small area where the resulting high intensity ionizes the air molecules (blue star) [1]. (**e**) Background reservoir effect on filamentation: fluorescence images of the filaments without and with a pinhole (white arrow) [30].

As depicted in Figure 2a–c, femtosecond laser filamentation is a unique nonlinear optical phenomenon that occurs during high-power ultrafast laser propagation in all transparent optical media (gases, liquids, and solids). During filamentation, a plasma channel, whose length can exceed the Rayleigh length, is generated. Due to the different free electron generation mechanisms, the obvious difference is the plasma density and the length of the filament for different gases and condensed matter materials. Meanwhile, a multitude of linear and nonlinear optical effects, such as diffraction, dispersion, Kerr self-focusing, plasma defocusing, supercontinuum generation, super clean fluorescence emission and amplification, and self-pulse compression, are involved. Generally, the complicated physical scenario of filamentation can be simply considered as the dynamic balance between Kerr self-focusing and self-generated plasma defocusing. Although a few excellent reviews on femtosecond laser filamentation are already available [1,9], here we will briefly summarize the physical mechanisms, exotic properties, and the ultrabroad spectra generation of the filaments that are induced by femtosecond laser in this section, toward filament-based sensing.

### 2.1. Physical Mechanism of Filamentation

The filamentation in transparent optical media of high-power femtosecond laser pulses involves a number of nonlinear effects such as diffraction, self-focusing, GVD, and plasma that are generated by multiphoton and tunnel ionization [15]. Based on the nonlinear wave equation and the retarded coordinate system $\tau =t-z/v_{g}$ ($v_{g}$ stands for group speed), the spatio-temporal dynamics of femtosecond laser during filamentation can be written as [1]:

(1) $$2ik_{0}\frac{\partial A}{\partial z}+(1-\frac{i}{\omega_{0}}\frac{\partial }{\partial \tau })\Delta_{⊥}A+2k_{0}DA+\mu_{0}\omega_{0}{}^{2}(1+\frac{i}{\omega_{0}}\frac{\partial }{\partial \tau })p_{NL}=0,$$

where A(z,r,τ) is the spatio-temporal envelope of the pulse, r stands for the transverse radius, $\omega_{0}$ indicates the central frequency, $k_{0}=n_{0}\omega_{0}/c_{0}$ is the linear propagation constant with $n_{0}$ as the liner refractive index, $c_{0}$ is the speed of light in a vacuum, $\mu_{0}$ is permeability of vacuum, $\Delta_{⊥}$ is Laplacian along transverse direction, D describes the high order dispersion, and $P_{NL}$ is the nonlinear polarization [16]. Alternatively, focusing on the electric field of the laser pulse rather than the pulse envelope, the Moloney group developed a unidirectional optical pulse propagation equation and corresponding numerical simulation method. This scheme can accurately calculate the instantaneous complex amplitude of an optical pulse, so that the computation is greatly increased and a higher computing platform is required [17,18,19].

The underlying mechanism of the filamentation has previously been recognized as the dynamic balance between self-focusing originating from the optical Kerr effect and self-defocusing that is induced by self-generated plasma during the nonlinear propagation of femtosecond laser pulses, which can be well described and simulated numerically by Equation (1). However, the intuitive physical scenario of filamentation has three alternative interpretations including the self-guiding model and the moving focus model. The self-guiding model [20,21] including diffraction, self-focusing, and plasma defocusing effect, is an extension of the self-trapping model [22]. The formation of filament in air was interpreted by a quasi-stationary balance scheme. According to this model, the laser pulses travel inside a self-induced wave guiding structure consisting of a weakly ionized inner core that is surrounded by a cladding. However, later experimental results suggested that the moving focus model that is based on a dynamic balance, seems to give a better description of the filamentation [23,24,25,26]. As depicted in Figure 2d, the collimated laser pulse with a transverse Gaussian distribution can be regarded as a series of successive slices in the temporal domain. The pulse slices will lead to an intensity-dependent Kerr nonlinear index $\Delta n_{k}$, and converge to the focal point providing the power is high enough to ensure that the self-focusing “lens” overcomes diffraction, that is, exceeds the critical power $P_{\mathrm{cr}}$ [27]. The self-focusing distance is:

(2) $$z_{\mathrm{sf}}=\frac{0.367ka^{2}}{\sqrt{{\left[(P/P_{\mathrm{cr}}{)}^{1/2}-0.582\right]}^{2}-0.0219}},$$

where k and a are the wave number and the radius of the beam profile at the 1/*e* level of intensity, respectively, and *P* is the peak power of the slice. It illustrates that the central slice has the highest power; thus, it will form the self-focus earliest at shortest distance; while lower power slices will encounter their foci later at longer distances. Therefore, the self-foci corresponding to different pulse slices leads to the perception of ‘filament’. Before the slices self-trap into a singularity, the laser intensity will be high enough to ionize the medium and produce a weak plasma density (~10^14^ to 10^16^ cm^−3^) [2,3,4,5,6] that defocus the focused slice back to background, forming an energy reservoir. The dynamic balance of the self-focusing and the plasma defocusing effect is reflected as the energy exchange between the filament core and the background energy reservoir during propagation (Figure 2d). The background reservoir is very critical for filamentation: the pinhole blocking the background and passing the central filament would immediately stop filamentation (Figure 2e) [28,29,30]. By applying the moving focus model, the observed beginning and end of the filament were successfully predicted by the theoretical calculations [24]. Although all the experiments noted that the tiny filament is embraced by a wide background energy reservoir, only the moving focus model could account for the observed small ratio of the energy that was contained within the filament zone to the total laser energy (~10%) [24].

### 2.2. Exotic Properties of Filaments

Owing to the exotic properties such as intensity clamping, mode self-cleaning, pulse self-compression, self-healing, and long-range filamentation, femtosecond laser filamentation provide more possibilities and opportunities for remote sensing under different complicated conditions.

#### 2.2.1. Intensity Clamping

**Figure 3:**
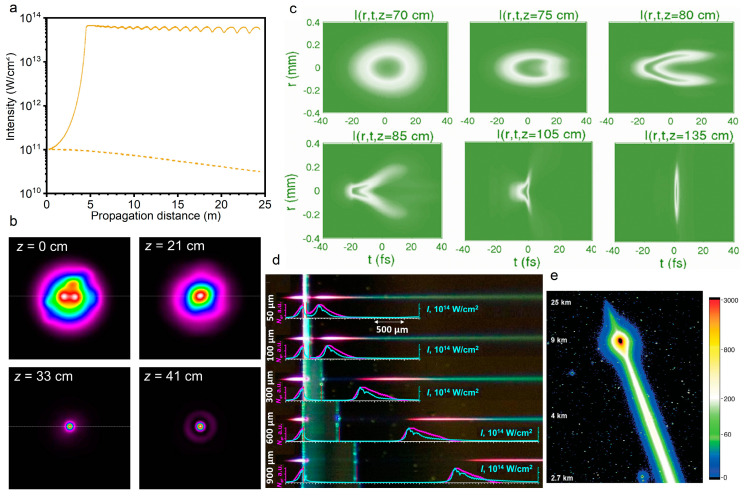
Exotic properties of filaments. (**a**) Self-focused intensity is clamped as a result of the generated plasma in the filament [20]. (**b**) Laser beam profiles at various propagation distances when the power is equal to three times the critical power for self-focusing in air [41]. (**c**) Pulse self-compression to the single-cycle limit by filamentation in a gas with a pressure gradient [53]. (**d**) Experimentally recorded femtosecond laser filamentation in a fused silica sample with different air gap widths. The laser pulse propagates from the left [61]. (**e**) Typical image of kilometer-range filamentation taken with the CCD camera of the Tautenburg observatory at fundamental wavelength and 1 s exposure time [63].

Filamentation arises from the dynamic balance between the optical Kerr effect that is induced laser beam self-focusing and the plasma defocusing effect of the self-generated weak plasma, that is, $\Delta n_{k}+\Delta n_{p}=0$ ($\Delta n_{k}$ and $\Delta n_{p}$ represent the refractive index changes that are caused by the Kerr effect and plasma effect, respectively). It will cause a limitation of the minimum diameter and the peak intensity during the laser pulse propagation, which is known as intensity clamping [12,13,31]. The intensity depends on the difficulty of generating plasma in the self-focal zone for material. It was demonstrated that the clamped intensity is 4 × 10^13^ W/cm^2^ for a laser pulse of 100 fs duration from theoretical to experimental observations (Figure 3a) [12,32]. Owing to the clamping of the peak intensity inside the filaments, a constant blue shift of the super-continuum spectrum can be expected for pulse energies above a threshold [33].

#### 2.2.2. Mode Self-Cleaning

The significant improvement of the beam quality was noticed by examining the far-field pattern of the conical emission surrounding a filament core. Specifically, the conical emission that is associated with the filament exhibits a single transverse mode while the residual incident pulse outside the filament presents a poorer beam quality [34,35]. It was demonstrated that the self-improvement of the beam quality can be attributed to the interaction between the self-focusing and the diffraction in the filament with an excellent energy stability in the core due to intensity clamping [34,36,37,38,39]. In detail, when the power is not too high so that multiple filaments are not yet induced, the intensity perturbation in the initial beam profile could be treated as the superposition of high order spatial modes on fundamental mode. Then, the self-focusing of the laser beam, acting as a spatial filter, focuses the fundamental mode toward the propagation axis while the self-focusing of higher order modes could not overcome diffraction, thus the fundamental mode was coupled and propagated in the self-focal region, resulting in the self-cleaning behavior of the pulse mode (Figure 3b) [40,41]. However, if the laser power is considerably higher than the critical power, the improvement of beam quality cannot be retained due to the emergence of multiple filaments. Such interesting mode self-cleaning effects in filamentation is promising for high beam quality tunable laser amplification.

#### 2.2.3. Pulse Self-Compression

In the filamentation process, the propagated femtosecond laser pulses undergo multiple self-action nonlinear processes including self-focusing, self-phase modulation, self-steeping, and splitting arising from group velocity dispersion. Higher order effects induced multiple splitting and coalescence of pulses, etc., thereby the spatio-temporal behaviors can be reshaped [42,43]. Besides the spatial self-cleaning, the temporal self-compression of pulse at normal GVD conditions is also fascinating [36], which provides a convenient technique to obtain the reproducible pulses with excellent beam quality and few cycles, especially attosecond pulses in the far UV, from longer pulses [44,45,46,47,48,49,50,51,52]. It was demonstrated theoretically that the pulse duration can be compressed to single optical cycle limit in a gas with an optimal pressure gradient by filamentation (Figure 3c) [53]. Then, the self-compression of 45 fs pulses down to below 8 fs duration without the need for any additional dispersion compensation was achieved by filamentation in noble gases [54]. Noticeably, self-phase modulation leads to spectral broadening and relative phase mismatching among the spectral components. The temporal profile and spectra of pulses evolve along the propagation path, therefore, there is a self-compression region and optimum position [55].

#### 2.2.4. Self-Healing

The high intensity (10^14^ W/cm^2^) and electron density (10^15^ cm^−3^) inside a filament modify the medium properties while propagating, which gives hope for a relative insensitivity to external condition variations [56]. Additionally, the size of the background energy reservoir is crucial to maintain the high-intensity filament core, which is about 5–10 times larger than the core and contains up to 50% of the pulse energy [30,57]. Intriguingly, relying on the energy replenishment from the background reservoir, filaments have an enough energy to recover filamentation and survive from the collisions with droplets (~100 μm in diameter), known as the self-healing or self-reconstruction of femtosecond light filaments (Figure 3d) [58,59,60,61]. This phenomenon provides an opportunity to penetrate through fog and clouds for laser beams, which is a key issue for free space laser communication, LIDAR detection of atmospheric pollutants, telemetry, range finding, and active imaging, etc. [56,62,63].

#### 2.2.5. Long-Range Filamentation

Long-range filamentation, femtosecond pulse delivering the laser energy over long distances without diffraction, is spectacular and attractive, especially for scientists who have remote sensing in mind. Compared with air and condensed matters, the free-electron generation process is different: air molecules are partially tunnel-ionized with weak plasma density (~10^14^ to 10^16^ cm^−3^) [2,3,4,5,6], whereas condensed matter are excited by inverse bremsstrahlung and partial cascade ionization with a plasma density of 10^18^ cm^−3^ [1]. Generally, the filament length in condensed matters is much shorter than in air due to significant pulse attenuation in plasma generation and subsequent interaction. Moreover, the filament core in air with an intensity threshold of 10^12^–10^13^ W/cm^2^ contains only about 10% of the pulse energy because of the intensity clamping and background energy reservoir. Therefore, atmosphere that the most accessible transparent medium provides a suitable environment for long-range filamentation. Up to now, a major effort and international collaboration have been devoted to long-range filamentation over several kilometers in air (Figure 3e) [63,64,65,66].

### 2.3. Ultrabroad Spectra Generation

Owing to the involved abundant nonlinear optical effects in femtosecond laser filamentation, the ultrabroad spectra emission covering ultraviolet, visible, infrared, and terahertz bands can be generated. The typical phenomena such as supercontinuum generation, air lasing, tunable ultrashort laser pulses, and terahertz emission have been extensively studied, which can provide more flexible schemes for filamentation-based sensing. Limited by space and theme, other important phenomena of ultrabroad spectra generation during filamentation, such as the generation of attosecond laser pulses and higher harmonic, will not be discussed here.

#### 2.3.1. Supercontinuum Generation

**Figure 4:**
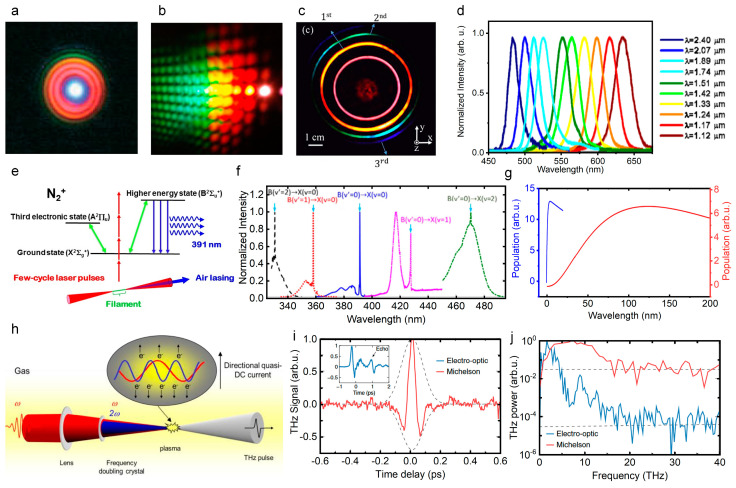
Ultrabroad spectra generation during filamentation. (**a**) Supercontinuum that is generated from femtosecond laser filamentation [11]. (**b**) Multi-color laser arrays that are generated in the sapphire crystals [82]. (**c**) Multi-color ring that is generated from the interaction between double femtosecond Bessel laser beams and silica [84]. (**d**) Tunable and stable ultrashort laser pulses in the visible spectrum are generated with high efficiency by a four-wave mixing process during the filamentation of near-infrared laser pulses in gases [39]. (**e**) Schematic image of the mechanism of the lasing action during filamentation in air where three electron states are involved [85]. (**f**) Spectra of N_2_^+^ lasing radiation that is excited by the pump laser with different wavelengths (from left to right: 1682, 1760, 1920, 2050, 1415 nm) [86]. (**g**) Calculated formation dynamic of the *C*^3^Π_u_^+^ state molecules that are based on the electron impact mechanism (blue line) and the dissociative recombination mechanism (red line) for 1 bar [87]. (**h**) Schematic of strong THz pulse generation from two-color laser-produced gaseous plasma [88]. (**i**,**j**) Temporal waveform and spectrum of THz that is generated by two-color laser field [89].

During filamentation, the nonlinear effects that are induced by the powerful femtosecond laser pulses such as self-phase modulation, four-wave mixing, and self-steeping can lead to the spectral broadening and generate supercontinuum (Figure 4a) [11,67], which is promising for spectroscopy, fluorescence microscopy, optical communications, remote sensing, and few-cycle pulses generation. Oriented by these applications, the spectral component and conversion efficiency of supercontinuum that is modulated by pulse energy, duration, chirp, polarization, filament length and pattern, and mediums have been brought into focus recently. Specifically, the spatial beam shaping of the incident laser by axicon [68], micro lens array [69,70], phase plate, and spatial light modulator [71] can redistribute the energy distribution and alter the quality parameters of filaments such as the length, pattern, and plasma density as required, thereby optimizing the supercontinuum generation in filaments. Besides, the temporal pulse shaping of femtosecond laser can effectively modulated the ultrafast dynamics of filamentation and greatly affect supercontinuum generation. In principle, almost arbitrary pulses in time can be obtained by controlling the amplitude, phase, and polarization of femtosecond laser pulses in the frequency domain through the typical pulse shaping system comprised by grating pair, lens pair, and phase modulator [72,73].

#### 2.3.2. Tunable Ultrashort Laser Pulses

Based on the four-wave mixing effect, ultrashort bandwidth radiation can be generated in the frequency range covering the ultraviolet band [74,75,76,77] to the infrared band and even the THz band [78,79]. The tunable ultrashort laser pulses correspond to a wide bandwidth and an ultrashort pulse duration (even femtoseconds). It was firstly demonstrated that the tunable ultrashort laser pulses that range from near-infrared to ultraviolet such as multicolor femtosecond laser array and ring (Figure 4b,c) can be generated from the nonlinear interaction between ultrashort lasers and nonlinear crystals [80,81,82,83]. Actually, the generation of tunable ultrashort laser pulse in air is more important for remote sensing. Owing to intensity clamping and self-filtering in the filament, the stable and high-quality visible laser pulses can also be produced by the four-wave mixing process during the filamentation of near-infrared laser pulses in gases (Figure 4d) [39,84]. Furthermore, the four-wave mixing process during the filamentation can produce the high flux tunable ultrashort pulses in gas medium without having to consider a damage threshold.

#### 2.3.3. Air Lasing

Air lasing is a concept that refers to remote no-cavity (mirrorless) optical amplification in ambient air with the air constituents as the gain media (Figure 4e,f) [85,86,90,91]. This was first proposed by Chin’s group from the observation of amplified spontaneous emission (ASE) in a femtosecond filament in 2003 [90]. The highly directional air lasing emissions have great prospects in the fields of atmospheric sensing and pollutant detection. Therefore, various pumping schemes have been proposed, and oxygen atoms, nitrogen atoms, nitrogen molecules, and nitrogen ions have been confirmed so far as gain media in air to build up population-inversion [85,86,91,92,93,94]. Based on the laser-induced fluorescence depletion technique, the population evolution dynamics of excited molecules can be measured with a femtosecond time resolution [87,95]. It has been demonstrated that the excited neutral molecules can be formed in an ultrafast timescale of a picosecond due to the electron impact excitation of the excited neutral nitrogen molecules inside filament (Figure 4g). Thus, the population dynamics of excited molecules depend on atmospheric pressure [87,95]. In contrast, the ions system emission such as the $C^{3}\Pi_{u}^{+}\to B^{3}\Pi_{g}^{+}$ transition of nitrogen ions $N_{2}^{+}$ demonstrates several unusual features, including ultrafast gain building, possible super radiant emission, and strong polarization dependence of pump laser [87,91,95,96,97,98,99,100]. However, the physical mechanism of such emission phenomenon is still controversial [91,98,101].

#### 2.3.4. Terahertz Emission

Femtosecond laser filamentation have been demonstrated as an ideal source for strong THz pulses. During intense laser-plasma interactions, the electrons are accelerated by the driving laser field, the terahertz emission can be generated due to the accumulation of the photocurrent [102,103,104], which can be well explained by transition-Cherenkov radiation model, ponderomotive force model, and bremsstrahlung model [88,89,102,105,106,107]. To further explain the strong spatial confinement of THz pulse by femtosecond laser filamentation, we have established a three-step procedure by combining the one-dimensional negative dielectric waveguide model with the conventional four-wave mixing and photocurrent models [108,109]. Note that the efficiency of THz generation in the filament that is produced by the single-color laser pulses is quite low. Therefore, various enhanced schemes such as multiple filaments, electrostatic field, and two-color laser field were improved [88,110,111,112,113], where the last one has been extensively considered as most effective method. During two-color laser filamentation, the fundamental wavelength laser pulse propagates through a nonlinear crystal to produce a second-harmonic laser pulse. The collinearly transmitted two-color laser is simultaneously focused to generate two-color filament (Figure 4h). The THz that is generated by the two-color filament significantly enhances the conversion efficiency, and its ultrabroad bandwidth spectrum is far beyond the electro-optic crystal and photoconductive antenna (Figure 4i,j). Furthermore, the THz field would reach several MV/cm with a driving laser energy of 15 mJ, which paves the way of THz nonlinear research [114,115]. Moreover, this collinear two-color field coupling method provides great convenience for THz coherent control [116,117].

## 3. Sensing and Controlling of Filamentation

Sensing and controlling of filamentation can lay a solid foundation for the filament-based sensing techniques and related applications. On the one hand, the sensing of filamentation, including characterizing the filamentary light pulse, the evolution of plasma, and the molecular or atomic excitation dynamic can deepen our understanding of the filamentation and provide insights into the strong-field–molecule interactions in sensing. On the other hand, the various spatio-temporal shaping techniques for femtosecond laser pulses can adjust and control the location, length, and plasma density of filament, facilitating that the filamentation-based remote sensing is performed according to our requirements.

### 3.1. Sensing of Filamentation

#### 3.1.1. Laser Intensity in Filament

**Figure 5:**
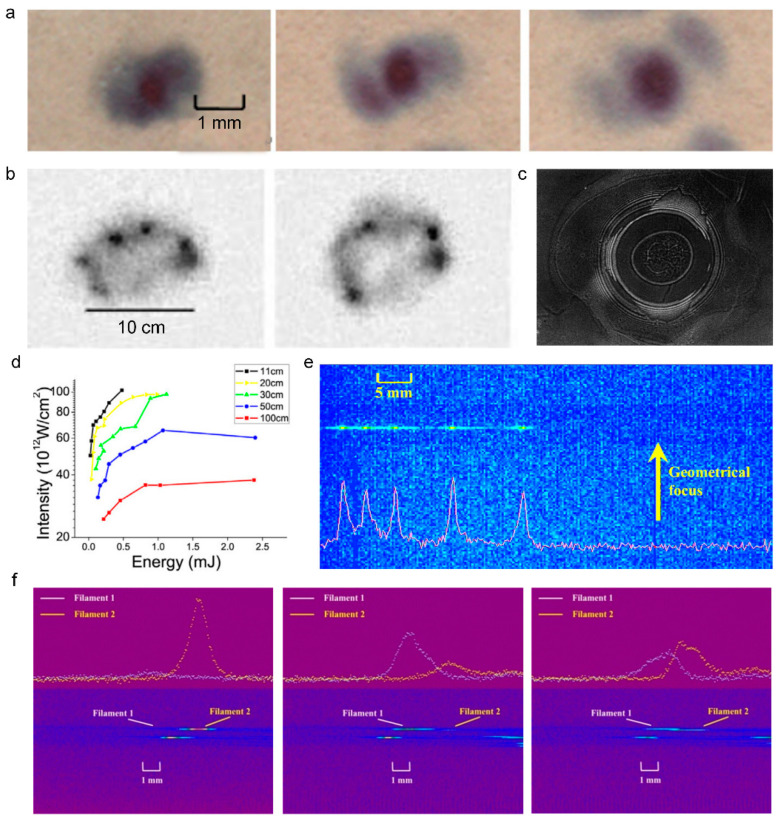
Sensing of laser intensity in filament. (**a**–**c**) Transverse laser intensity profile by exposure of (**a**) burn paper [121], (**b**) photographic plates [120], and (**c**) ablation of silica glass plates [122]. (**d**–**f**) Measurement of laser intensity by fluorescence of filament: (**d**) laser peak intensity as a function of input laser energy [123]; experimental observation of (**e**) multiple self-focusing [124] and (**f**) multifilament competition [10].

As the intensity in filament core is high enough to damage almost all the detectors, the direct invasive measurement of filament is very difficult. Utilizing the successive glass plates that are placed in the grazing incident angle to attenuate the laser intensity, the spatial profile of filament can be obtained by a detector [118,119]. Additionally, considering the complex evolution of pulse inside the filament, several related physical effects were adopted to deduce the laser intensity indirectly. It was demonstrated that the transverse laser intensity profile can be recorded by the exposure of photographic plates [120], burn paper [121], and the ablation of silica glass plates [122]. Figure 5a depicts the laser profiles that were measured by burn paper at different propagation distances, corresponding to the beginning (z = 95 cm), the middle (z = 110 cm), and the end (z = 130 cm) of the filament. Figure 5b shows the laser intensity profiles that were obtained by photographic plates at a long distance of 830 m that were recorded at different laser shots. Figure 5c presents the transverse laser intensity profile by ablation of silica glass plates.

Moreover, the in situ, noninvasive methods were also presented based on the high energy cutoff of harmonics [32] and the fluorescence intensity [10,123,124]. The fluorescence method is adaptable to visualize the filament intensity along the filament at a remote distance. Due to the difference of the excitation mechanism, the intensities of different fluorescence emission spectral lines present different power law relationships with laser intensity. Consequently, based on the signal ratio (R) of two nitrogen fluorescence at 391 nm and 337 nm, an empirical formula was established to estimate the laser intensity for filamentation in air. Figure 5d shows the laser peak intensity under the different laser energy was deduced by the empirical equation. The multiple refocusing [124] (Figure 5e) and the multiple filament competition dynamics [10] (Figure 5f) were directly observed by such a fluorescence method.

#### 3.1.2. Plasma Density in Filament

**Figure 6:**
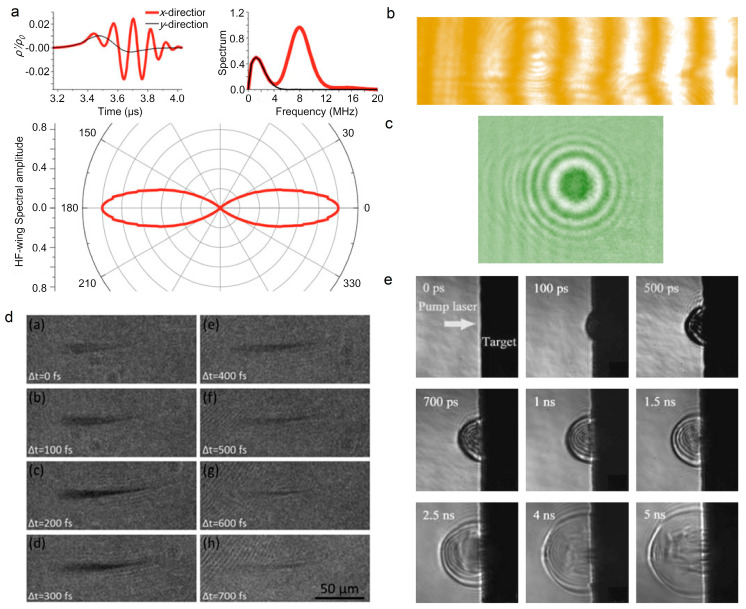
Sensing of plasma density in filament. (**a**) Acoustic signals that are induced by plasma grating [127]. (**b**) Interferometric images for femtosecond laser that are induced plasma in fused silica [128]. (**c**) Longitudinal diffraction fringe [119]. (**d**,**e**) Time-resolved shadowgraphs: (**d**) transient plasma that is induced by spatiotemporally focused femtosecond laser inside fused silica [129]; (**e**) material ejection during the ablation of an aluminum target [130].

The electronic density inside the filament is a crucial parameter for characterizing and controlling the filament. It also benefits the application in remote sensing and lightning discharge guidance. The electrical conductivity is directly related to the electronic density [4], while it is low-accuracy. The signal intensity of acoustic waves that are emitted from filament is also used to determine the electronic density, while a supplementary calibration is needed [125,126]. The axial plasma profile can be obtained by monitoring the acoustic waves. Figure 6a shows the waveform, spectra, and the directional distribution of acoustic signals that are induced by the plasma grating [127].

Additionally, the plasma density evolution can be obtained by various pump-probe techniques, including femtosecond interference microscopy [131,132], interferometric fringe imaging [3,7,128] (Figure 6b), longitudinal diffraction [15,133] (Figure 6c), and shadow imaging [8,129,130,134] (Figure 6d,e). The methods of interference and diffraction are based on the phase shift resulting from the plasma in filament. Combined with a linearly chirped probe laser pulse, the longitudinal diffraction method can be performed to investigate time-resolved ultrafast ionization dynamics. The shadow imaging depends on the absorption effect of the plasma. The absorption coefficient α depends on the imaginary part of the refractive index κ and the speed of light *c*, α=2ωκ/c. The complex refraction index $\tilde{n}=n+i\kappa$ can be deduced by the dielectric function according to the Drude model:

(3) $$\tilde{\varepsilon }=1-\omega_{p}^{2}\left[\frac{\tau^{2}}{\left(1+\omega^{2}\tau^{2}\right)}+i\frac{\tau^{2}}{\omega \tau \left(1+\omega^{2}\tau^{2}\right)}\right],$$

where *τ* is the scattering time, and $\omega_{p}=\sqrt{e^{2}N_{e}/m_{e}\varepsilon_{0}}$ is the plasma frequency that is related to the plasma density *N*_e_ [135]. The dynamic process of plasma formation (Figure 6d) and material processing (Figure 6e) by femtosecond laser can be recorded by time-resolved shadowgraphs. Moreover, the plasma density is proportional to the Stark broadening line width of atoms that are inside the filament, therefore the atomic spectroscopic analysis is a simple and effective method [136]. The THz spectroscopy can be also adopted to measure the plasma density inside the filament, which determines the peak frequency of the THz wave that is emitted from the filament [137].

#### 3.1.3. Laser Pulse in Filament

Due to group velocity dispersion, self-steepening, pulse splitting, space-time focusing, etc. in filament, the femtosecond laser pulse experiences extreme and complicated spatio-temporal distortion. It is important to investigate the temporal evolution of a pulse during filamentation for fundamental filament research, spectral analysis, pulse compression and attosecond pulse, and high-order harmonics generation. Although the direct measurement of the laser pulse evolution in the time domain has been limited by the high clamping intensity inside the filament, conventional methods, such as autocorrelation, FROG (frequency-resolved optical gating) and SPIDER (spectral phase interferometry for direct electric-field reconstruction), can also be used to measure the pulse shape inside the filament by grazing incidence in a glass plate [54]. However, the measurement result of these invasive methods would be influenced by the dispersion and nonlinear effect of the glass plates. 

**Figure 7:**
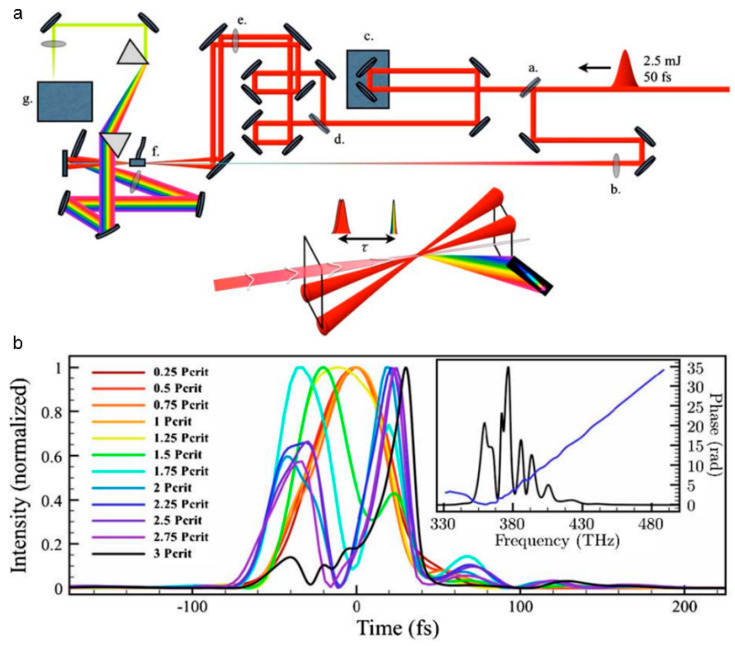
Sensing of laser pulse in filament. (**a**) Experimental arrangement and (**b**) filament temporal profiles for the TG-FROG measurements of filaments under different laser power. a. 80/20 beam splitter; b. *f* = 2.07 m lens; c. delay stage; d. 50/50 beam splitter; e. *f* = 0.5 m lens; f. argon jet; g. USB spectrometer. Pcrit: critical power for self-focusing [141].

In situ methods have also been presented, such as two-photon fluorescence [138,139], Raman scattering [140], and transient-grating cross-correlation frequency-resolved optical gating (TG-FROG) [141]. Specifically, the pulse duration and chirp parameters along the filament can be accessed by two-photon fluorescence. The Raman scattering method is based on the rapid temporal vibrational Raman response of molecules such as H_2_O, N_2_, and O_2_ in the medium, which is appropriate for measuring the self-shortened filament pulse. Both the temporal phase and amplitude can be obtained by TG-FROG (Figure 7a). The temporal pulse self-steeping, splitting, and compression were observed during filamentation by TG-FROG, as displayed in Figure 7b.

### 3.2. Controlling of Filamentation

The femtosecond laser filamentation exhibits unique advantages in remote sensing, THz generation, laser precision processing, pulse compression, etc. Depending on the different demands of applications, the controlling of filament is necessary, such as the spatial profile of laser intensity, including the spatial position, filament length, laser intensity, plasma density, and the organization of the multiple filaments.

#### 3.2.1. Spatial Beam Shaping

**Figure 8:**
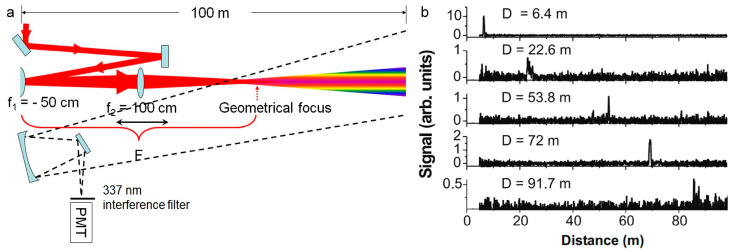
(**a**) Telescope system for generating long-distance filament, where PMT stands for photomultiplier tube. (**b**) Collected backward fluorescence signals at different distances [142].

The spatial controlling of filament mainly refers to changing the focal length to control the filament position and adjust the laser intensity. Figure 8a shows the telescope system, which is always used to control the position of the filament by changing the distance between the negative and positive lens [142]. The combination of telescope system and the backward detection system in Figure 8a is generally used to collect the fluorescence signals at certain distances (Figure 8b) in remote sensing. As mentioned above, the dynamic balance between the Kerr self-focusing effect and the plasma defocusing effect leads to the intensity clamping effect. Improving the intensity in the filament core is valuable to enhance the signals in remote sensing, and improve the conversion efficiency of high-order harmonics. It has been confirmed that the external tight focusing condition plays an important role in increasing the clamping intensity [143]. The length and diameter of the plasma channels are also external focusing-dependent. Specifically, the plasma density (*f* = 10 cm) is more than three orders of magnitude higher than that of *f* = 380 cm [15]. However, under tight focusing conditions, the laser filament cannot be launched far away, which makes it difficult to realize remote sensing. Under large numerical aperture (NA) conditions, optical breakdown emerges and coexists with filamentation [144]. The competition between filamentation and optical breakdown is significant in the application of waveguide writing and femtosecond laser surgery for myopia.

**Figure 9:**
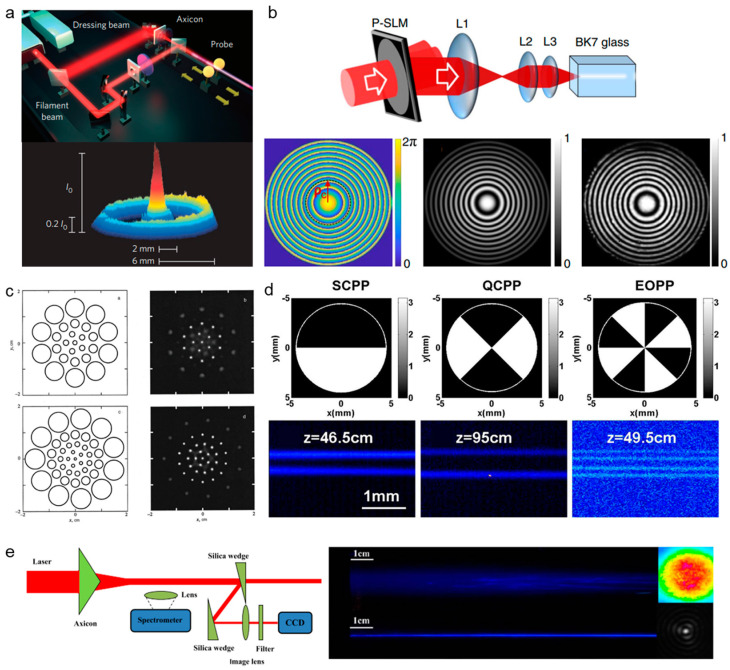
Controlling of multiple filaments by (**a**) an auxiliary beam [148], (**b**) phase-only spatial light modulator (P-SLM) [153], (**c**) lens array (left: lens arrays; right: the filaments that were formed by corresponding arrays) [155], (**d**) phase plates (SCPP, semicircular phase plate; QCPP, quarter-circle phase plate; EOPP, eight-octant phase plate) [157], (**e**) axicon [146].

Due to smooth beam profile with only one intensity maximum, it is difficult to realize in the real experimental condition, the multiple filaments that are often observed when the laser peak power is higher than the critical power for self-focusing [10]. Hence, multiple filament management by spatial beam shaping is an important part in manipulating filaments. The controlling of multiple filaments includes suppressing and organizing multiple filaments. The generation of multiple filaments is sensitive to the relative distance of the initial perturbations. Therefore, reducing the beam diameter by the telescope system in Figure 8a, the formation of multiple filaments can be suppressed effectively [145]. Reshaping the phase of the laser beam can also restrain multiple filaments by using axicon [146], π phase plate [119], and spatial light modulator [147], as a result of the reduced energy around the hot spot. An auxiliary beam is also used to suppress multiple filaments and extend the filament length [148], as shown in Figure 9a. In remote sensing, the backward fluorescent signal fluctuates on a shot-to-shot basis, due to the competition among multiple filaments [149]. By suppressing multiple filaments, the backward fluorescent signal becomes more stable for remote sensing [145].

For high a peak power laser pulse (~TW), it is unavoidable for the laser beam to split into multiple filaments [150]. Disordered multiple filaments limits the applications in writing waveguides [150], microwave guiding [151], and terahertz generation [152]; how to organize the formation of multiple filaments becomes an important research topic. It is effective to control multiple filaments by modulating the intensity disturbance and separating the background energy reservoir, based on spatial light modulator [153,154] (Figure 9b), lens array [155] (Figure 9c), phase plate [156,157] (Figure 9d), axicon [146] (Figure 9e), adaptive deformable mirror [66,158,159,160], metal mesh mask [161], changing the beam ellipticity [162,163,164], and gating [165], etc. [166].

#### 3.2.2. Temporal Pulse Shaping

**Figure 10:**
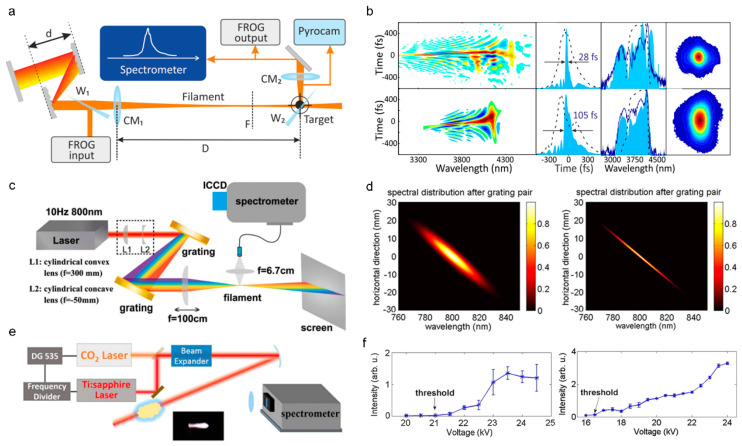
(**a**) Experimental setup for controlling the chirp to generate filament: CM_1_, curved mirror, assisting filamentation, *f* = 7 m; CM_2_, reimaging curved mirror; W_1,2_, attenuating CaF_2_-wedges; D, the distance between CM_1_ and the position of a “Target”; F, the position of the linear focus. (**b**) The compressed temporal pulse inside the filament [167]. (**c**,**d**) Experimental setup for improving the peak intensity of filament by spatiotemporally manipulating and the reshaped spatiotemporal distribution [174]. ICCD, intensified charge coupled device. (**e**) Experimental setup for enhancing the plasma density assisted by a nanosecond laser. DG 535: digital delay generator. (**f**) Compression of signal intensity using a single CO_2_ laser (left) and the dual laser beam (right) [180].

As shown in Figure 10a, introducing chirp to a laser pulse is efficient to manipulate the intensity profile of a filament [167]. When laser pulses with an initially negative chirp propagating in medium with normal dispersion, the pulse compression will improve the intensity in the filament (Figure 10b) [168,169], which is critical for various effects and applications of filamentation. For example, the intensity of THz wave that is generated from filament is sensitive to the initial chirp of a laser pulse [170,171,172]. Additionally, spatiotemporal manipulation was presented to improve the peak intensity in the filament core [173,174] and control the intensity of supercontinuum [175]. A more intense and shorter filament can be generated by spatiotemporally focusing a dispersed laser pulse [176,177]. In Figure 10c,d, different spectral components overlap less in space after passing through the cylindrical lens and grating pairs. Then, different spectral components are converged and chirp-free in focus. For remote sensing, the spatiotemporally manipulation will improve the spatial resolution and the fluorescence intensity. Moreover, the spatiotemporal manipulation method was also presented to control the intensity by combining the chromatic focusing system with chirped laser pulses [178].

Besides, optimizing pulse train in time can greatly affect the filamentation and enhance the plasma density in filament, which is also an important parameter for filament-based applications. The main method to manipulate the plasma is to introduce a longer laser pulse. The lifetime of plasma inside a filament is several nanoseconds. The plasma that is generated by a femtosecond laser will be heated by the succeeding nanosecond laser [179,180]. As shown in Figure 10e,f, by adding a delayed long pulse laser (i.e., ns laser), the lifetime of plasma can be prolonged by ten times [179], and the fluorescence intensity in remote sensing can be enhanced observably [180].

## 4. Sensing Technologies Based on Filamentation

Recent progress in ultrafast laser filamentation shows that under certain power and focusing conditions, femtosecond laser pulses can travel a long distance (>10 km) and produce an extended super illumination area, and its emitted light spectrum can cover the whole visible light range from ultraviolet to infrared [181]. As a result, a series of versatile sensing technologies that are based on ultrafast laser long distance filamentation have been generated.

### 4.1. Filament-Induced Plasma Spectroscopy

Owing to the advantages of high sensitivity, non-intrusiveness, and real-time, laser-based spectroscopic techniques including differential absorption LIDAR, laser absorption spectroscopy, and especially laser-induced breakdown spectroscopy (LIBS) have been extensively used for remote sensing trace species. Compared with other analytical methods, the LIBS technique has a significant advantage in preparing sample for in situ, remote, and stand-off analysis. Specially, the fluorescence of solid-, liquid-, and gas-phase targets with little or no sample preparation can be emitted from the plasma and contains quantitative information on the composition of the sample in the region of the breakdown [182,183]. The laser with pulse energy of tens of millijoules and a duration of a few nanoseconds was frequently used for the LIBS technique. The peak power ranging from 1 to 100 MW was focused on a sample with a diameter of few tens micrometers that can produce an energy fluence fluxes on the order of 10^10^ to 10^12^ W/cm^2^. However, the achievable detection distance of LIBS that was performed with nanosecond pulses is tens of meters, and the line emissions from species are frequently accompanied by plasma continuum emission during this breakdown due to the temporal overlapping of these emissions.

The advances in high-intensity femtosecond laser technologies have provided a more versatile and remarkable method for sensing with high sensitivity through femtosecond laser filamentation. As mentioned above, the clamped intensity of 5 × 10^13^ W/cm^2^ inside the filament is strong enough to dissociate or ionize gas molecules, to explode fine particles (dusts and aerosols) and to induce “rapid or partial” breakdown on solid targets, resulting in “fingerprint” fluorescence of molecular fragments [184,185]. Moreover, the quality, location, and length of filaments can be effectively controlled to meet the requirements for identifying a target and locating its position in principle, and the filamentation at a distance of few kilometers in the atmosphere was achieved. To further increase the operation distance and obtain a clean fluorescence, the femtosecond laser filamentation provides new opportunities for LIBS to meet the urgent needs of detecting and identifying chemical constituents of gases, vapors, solids, and aerosols in various scenarios. Therefore, remote filament-induced plasma spectroscopy (FIPS) performed with intense femtosecond laser pulses has been extensively applied to remote sensing in the past two decades. Compared with the complete ionization that is induced by nanosecond pulses in LIBS, the femtosecond laser induces the partial ionization of medium, exhibiting quite different ionization scenarios and spectral properties. Here, the title of FIPS is used, which is distinguished from the “breakdown” corresponding to complete ionization.

**Figure 11:**
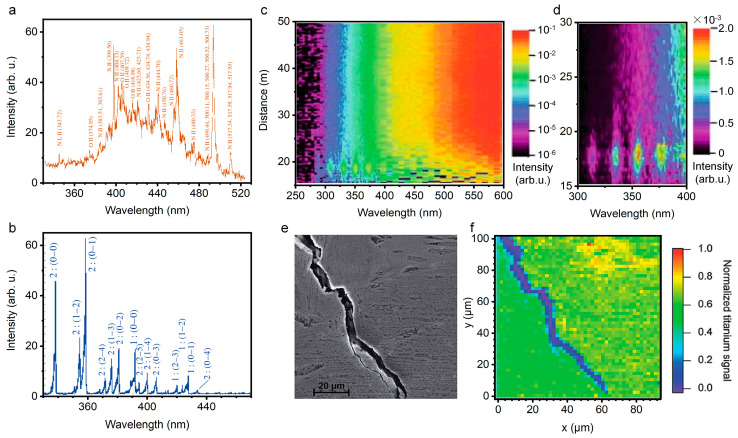
Filaments that were induced plasma spectroscopy. (**a**,**b**) Emission spectra of air in atmospheric pressure interacting with a Ti:Sapphire laser pulse of duration of 0.2 ns (**a**) and 200 fs (**b**) [187]; (**c**,**d**) intensity distribution of backscattered signal (**c**) and nitrogen fluorescence bands (**d**) from the filament as a function of the distance and the wavelength from filament [188]; (**e**,**f**) SEM graph (**e**) and FIPS map (**f**) of Ti–Al alloy surface [189].

Firstly, systematic, theoretical, and experimental studies have demonstrated that the “clean” fluorescence emissions of all targets that have been tested so far, ranging from gases, vapors, aerosols, to solids, can be obtained from the disturbance of excitation laser, super continuum, and even background light. Figure 11a,b show the emission spectra of air in atmospheric pressure interacting with a Ti:Sapphire laser pulse of duration of 0.2 ns and 200 fs. When the femtosecond pulse is focused in the atmosphere, the multiphoton or tunnel ionization dominates the plasma generation, rather than inverse bremsstrahlung and cascade ionization, due to the pulse duration being shorter than the averaged electron collision time in general conditions. Accordingly, the plasma density (10^15^–10^18^ cm^3^) and temperature (5000–7000 K) in the filament are much lower than that in the plasma that is induced by the common nanosecond-pulse, illustrating a lower background blackbody continuum [11,15]. Thus, the states of molecules can be clearly observed by using filament-induced fluorescence spectroscopy. Moreover, backward time-resolved spectroscopy from filament-induced by ultrafast intense laser pulses by time-resolved technique (such as controlling the delay time relative to trigger the signal and temporal gate width of ICCD equipped with spectroscopy) due to the fluorescence lifetime at the nanosecond level, the interference of background light can be further eliminated [186].

Besides, the well-developed LIDAR technique could deliver the laser pulses over long distances (up to kilometers scale) and generate powerful filaments. By using intense femtosecond laser pulses and LIDAR technique, the distance-resolved spectral intensity distribution of the backscattered light from long filaments can be achieved. Figure 11c,d depict the intensity distribution of the backward emission of the nitrogen fluorescence bands from the filament as a function of the distance and the wavelength. Clearly, filament-induced fluorescence spectroscopy can be used for identifying various substances in the atmosphere, including chemical and biological species ranging from gases and aerosols to solids [158,190,191,192,193]. Apart from the longitudinal spatial resolution, the strong confined laser plasma inside the filament can provide a quite high transverse spatial resolution. That is, chemical mapping (the distribution of a component) across a sample surface can be obtained through a filament scanning. For example, the FIPS-based chemical mapping was performed to measure micro-cracks or defects in Ti-Al alloys. As shown in Figure 11e,f, it can be concluded that FIPS mapping can be applied to identify the crack on the scale of several micrometers by comparing scanning electron microscope (SEM) and FIPS map in the selected region [186]. Similarly, considering the advantages in long-range propagation, remote imaging for the morphology and specific element distribution of a target object with a high spatial resolution can be achieved [194,195].

### 4.2. Filamentation-Based White-Light LIDAR

In recent years, environmental pollution and the greenhouse effect have become more and more serious. It is necessary to detect the chemical and kinetic processes of ozone pollutants in the troposphere and ozone layer destruction and loss in stratosphere by atmospheric remote sensing technology. With the development of ultrashort pulse laser technology, under the dynamic balance of diffraction, dispersion, the Kerr effect, and multi-photon ionization, femtosecond laser pulse can form a long self-guided optical filament structure, and accompanied by a narrow plasma channel, white light LIDAR technology that is based on this has been rapidly developed. The principle of an atmospheric LIDAR system is based on the optical scattering effect of emitted light on atmospheric components [196]. Compared with the traditional LIDAR, which is limited by the wavelength of narrow-band laser, the strong back component of white light supercontinuum spectrum [197,198,199] that is produced by femtosecond laser during long-distance filament formation makes the femtosecond LIDAR have the ability to perform high-resolution measurement at an ultralong distance in a wide spectral range [187,200].

**Figure 12:**
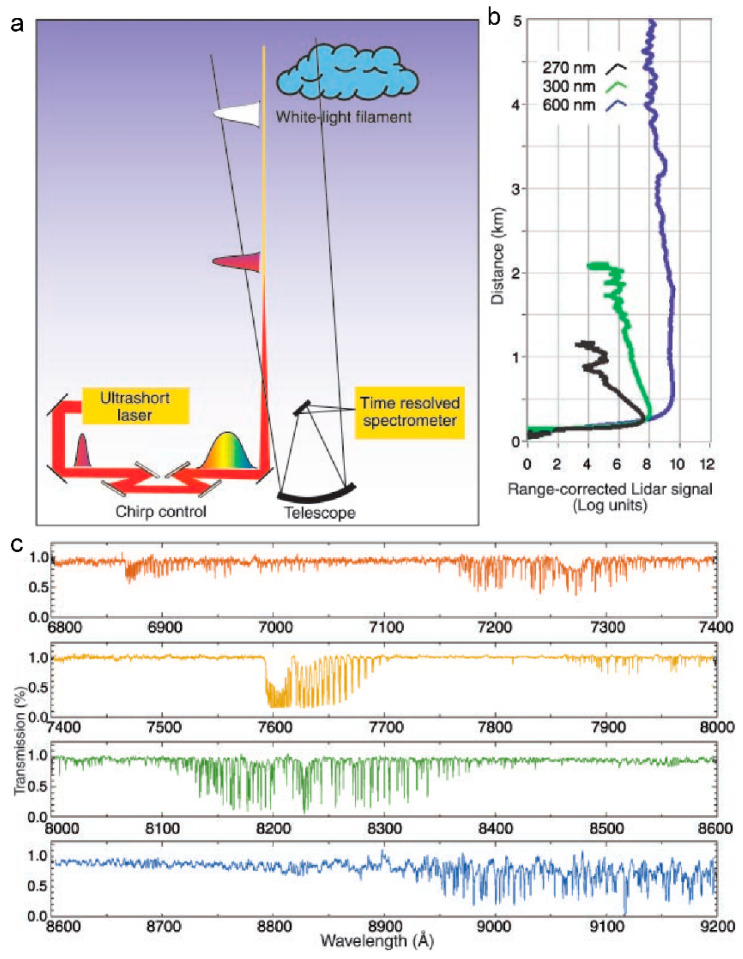
Filamentation-based white-light LIDAR. (**a**) Schematic of the LIDAR experimental setup. Before launch into the atmosphere, the pulse is given a chirp, which counteracts GVD during its propagation in air. Hence, the pulse recombines temporally at a predetermined altitude, where a white-light continuum is produced, and then is backscattered and detected by LIDAR. (**b**) Vertical white-light LIDAR profile at three wavelengths: 270 nm (third harmonic), 300 nm, and 600 nm. (**c**) High-resolution atmospheric absorption spectrum from an altitude of 4.5 km measured in a LIDAR configuration [201].

The atmospheric LIDAR system that is based on femtosecond laser filament-induced supercontinuum was put forward by Wöste et al. [201]. Then, Rairoux et al. [202] studied the propagation characteristics of TW-level ultrashort laser beam in the atmosphere. Using the generated supercontinuum laser combined with radar technology and time-resolved absorption spectroscopy technology, the absorption spectra of oxygen molecules and water vapor were measured. Kasparian et al. [63] reported the application of a supercontinuum laser that was generated by filament in the detection of atmospheric environment from several kilometers to tens of kilometers. As shown in Figure 12a, the femtosecond laser pulse is emitted into the atmosphere after passing through the (negative) chirp generator and becomes a filament at a predetermined distance. The white light that is backscattered by the filament is collected by the telescope and focused by the spectrometer on the time-resolved detector, so as to obtain the absorption spectrum of atmospheric molecules [201]. Different wavelengths of laser (270, 300, and 600 nm) have different transmission losses in the atmosphere (Figure 12b). The reason is that the laser with a shorter wavelength has stronger Rayleigh scattering, and the laser with wavelength of 600 nm can obtain high resolution atmospheric absorption spectrum at a height of 4.5 km (Figure 12c). In addition, the Doppler frequency shift that is generated by the backscattered echo signal can also be measured and calculated, and the radial wind speed (horizontal and vertical directions) and wind shear data in multiple altitude areas can be obtained.

### 4.3. Filamentation-Assisted Terahertz Remote Sensing

**Figure 13:**
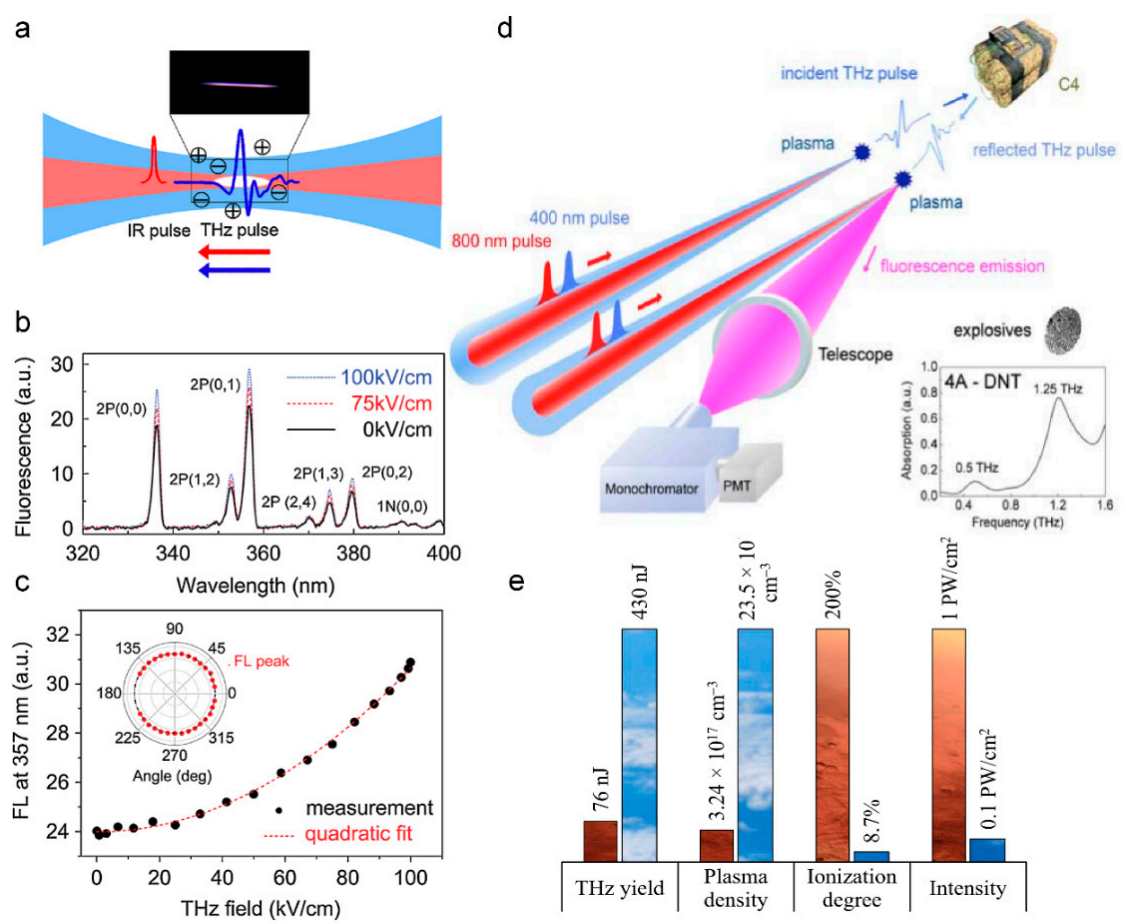
Filamentation-assisted terahertz remote sensing. (**a**) Schematics of the interaction between the THz wave and laser-induced plasma. (**b**) Measured fluorescence spectra versus the THz field at *t_d_* = −1 ps. Major fluorescence lines are labeled. *t_d_*: time delay between the THz pulse peak and the laser pulse peak. (**c**) Measured quadratic THz field dependence of 357 nm fluorescence emission line at *t_d_* = −1 ps. Inset: the isotropic emission pattern of THz-REEF [204]. (**d**) Envisioned scheme for THz stand-off generation and detection. There are two dual-color pulses that are focused close to the target under investigation creating a plasma emitter and a plasma sensor. For this figure, the absorbance spectrum of the target is retrieved through THz-REEF [209]. (**e**) The comparison of THz yield and femtosecond optical breakdown parameters in the atmosphere of Mars and Earth [210].

A terahertz wave lies between visible light and microwave in the electromagnetic spectrum, and its broadband, penetrability, and non-invasive characteristics are suitable for the characteristic spectrum detection and biomedical imaging of materials. However, the strong absorption of water vapor in the environment limits the long-distance transmission and detection of the terahertz wave. The fluorescence signal of nitrogen that is excited by femtosecond laser filamentation is of great help to terahertz remote sensing technology [203]. Zhang et al. proposed the THz fluorescent radiation enhancement technology (THz-REEF—THz radiation-enhanced emission of fluorescence), the principle of which is shown in Figure 13a–c [204]. The electron dynamics in the process of femtosecond laser-induced plasma is determined by the amplitude and phase, delay, and gas density of the laser pulse and terahertz pulse. The terahertz electric field can accelerate free electrons to make their collision more intense, so as to enhance the fluorescence emission of molecules or ions in the nanosecond time scale. The fluorescent signal of nitrogen that is excited by femtosecond laser filamentation in the air is in the ultraviolet band, while the ultraviolet fluorescent signal propagates in the air with high transparency and is not affected by the absorption of water vapor in the environment. That is to say, by using the characteristic that the terahertz electric field enhances the fluorescence signal omnidirectionally, the time-resolved enhancement effect of the fluorescence signal can be detected in a long distance, so that the time-domain information of the terahertz wave can be obtained reversely [205,206].

In addition, the omnidirectional emission of fluorescent signals that are excited by filaments also expands the application scenarios of terahertz detection technology. When terahertz wave is used to detect explosives and other dangerous goods, the intensity of terahertz wave that is generated based on photoconductive antenna or electro-optic crystal is low and cannot be transmitted or detected at a long distance. A terahertz wave that is generated by four-wave mixing in femtosecond laser filamentation must be measured in the forward direction, which limits the detection range [207]. The terahertz fluorescence radiation enhancement technology can solve this problem. Zhang et al. detected the time-domain signal of the terahertz wave at a distance of 30 m using THz-REEF, and obtained the characteristic absorption spectrum of 4A-DNT (4-amino-2,6-dinitrotoluene), as shown in Figure 13d [208,209].

Besides, compared with the pulse terahertz source that is generated by the traditional photoconductive antenna and electro-optic crystal, the energy of the terahertz wave radiated by two-color femtosecond laser filament in gas is stronger and there is no material damage threshold. The intensity of the terahertz wave that is generated by this scheme is related to the pressure of gas. Solyankin et al. simulated the comparison between CO_2_ gas pressure on the surface of Mars and the atmospheric pressure on the Earth, and radiated terahertz waves in CO_2_ gas with different pressures through the two-color field filament, as shown in Figure 13e [210]. Terahertz production near the surface of Mars is only six times lower than that on Earth, and the Martian atmosphere has a very low water vapor content (~0.03%). Therefore, a terahertz wave has low long-distance transmission loss on the surface of Mars, and can be used as a remote sensing technology for trace gases in the atmosphere of Mars.

### 4.4. Filament-Driven Impulsive Raman Spectroscopy

**Figure 14:**
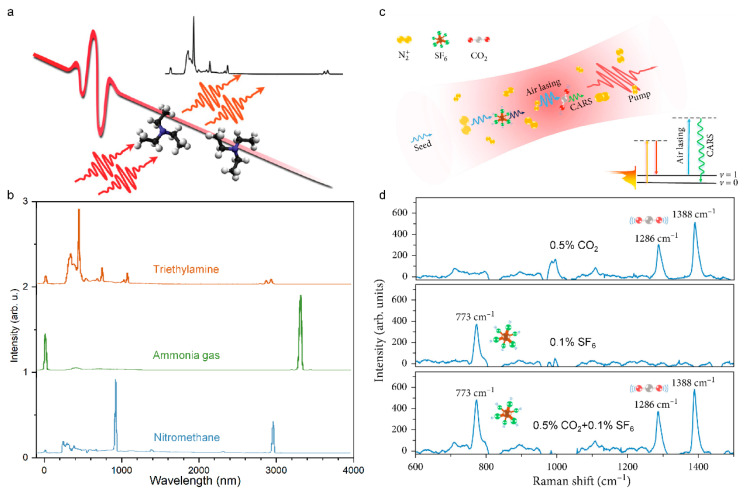
Filament-driven impulsive Raman spectroscopy. (**a**) Schematic diagram of filament-driven impulsive Raman spectroscopy. (**b**) Raman spectra of triethylamine, ammonia gas, and nitromethane that was measured using filament-assisted impulsive Raman spectroscopy [220]. (**c**) Schematic diagram of the basic principle for the greenhouse gas detection with air-lasing-based Raman spectroscopy. CARS, coherent anti-Stokes Raman scattering. (**d**) CARS spectra that was measured in the mixture of standard air and 0.5% CO_2_, 0.1% SF_6_, 0.5% CO_2_, and 0.1% SF_6_ [225].

Raman spectroscopy is an analytical method that is based on the Raman scattering effect [211,212,213] that was discovered by Indian scientist C.V. Raman to analyze the spectrum of scattered light with different frequencies from incident light, so as to obtain molecular vibration and rotation information, which can be applied to the study of molecular structure [214]. The scattering cross-section and molecular density of gas molecules are low, and the spatial distribution of the Raman scattered light is isotropic, which makes it difficult to perform spontaneous Raman spectroscopy detection under atmospheric pressure conditions [215]. Coherent Raman scattering (CRS) [216,217] signals occur in directional beams, and the collection efficiency is higher. However, CRS needs experimental components such as optical parametric amplifier (OPA) or hollow fiber (HCF) and chirp pre-compensation devices to produce the necessary tunability or bandwidth [218,219]. These components reduce the output pulse energy somewhat, thus reducing the maximum intensity of the remote detection point. Impulsive-stimulated Raman scattering (ISRS) can excite the whole vibration spectrum of the propagation medium by using the inherent pulse self-compression phenomenon that is generated during the filamentation of a femtosecond laser, thus realizing the remote detection of gas molecules by Raman spectroscopy (Figure 14a). Then, the ISRS technology was adopted to detect the Raman spectra of chloroform, dichloromethane, cyclohexane, toluene, pentane, triethylamine, ammonia, nitromethane, and gasoline at a distance of 2.5 m from the laser source [220], as shown in Figure 14b. In addition, the Raman spectra of ozone, nitric oxide, and nitrogen dioxide that are produced by corona discharge and chemical reaction in filament are detected by filament-assisted pulsed Raman scattering spectroscopy [221], which can be used for the classification of radioactive substances.

Unlike spontaneous Raman scattering, ISRS introduces femtosecond laser pulses to excite the vibration or rotation of molecules so as to improve the signal intensity [222]. But its difficulty lies in accurately controlling the time delay of pumping and detecting pulses at a long distance [223]. Ni et al. proposed that the pulse rotating Raman scattering of neutral nitrogen molecules can be realized by using the self-induced “air laser” that is generated during the filamentation of the femtosecond laser. The self-induced air laser can provide a strong laser pulse with a narrow bandwidth (<10 ps) as a detection pulse, so that the pump pulse and the detection pulse naturally overlap in time and space [224].

However, research was conducted in pure or high concentration gas molecules. Femtosecond laser filamentation inevitably leads to problems such as a poor signal-to-noise ratio and large signal fluctuation. It is still a challenge to improve the sensitivity and stability of ultrafast spectrum that is based on filament. To solve this problem, a highly sensitive air laser-assisted CARS technology was proposed [225,226], which uses an external seed amplification mechanism to improve the intensity of the N_2_^+^ laser and the signal-to-noise ratio of Raman scattering, and uses an orthogonal polarization arrangement to suppress the supercontinuum background. The technology can be used to detect SF_6_ greenhouse gases with concentrations as low as 0.03% in the air and to identify the isotopes of ^12^CO_2_ and ^13^CO_2_. The distribution of CO_2_ and its stable carbon isotope (^13^CO_2_) has regional differences. The accurate detection of its concentration change can reflect the impact of natural and human activities on the atmosphere in different regions.

## 5. Physical Mechanism of Femtosecond Laser-Induced Plasma

In the past decades, the FIPS has attracted more extensive attention than others filament-based sensing techniques. Therefore, here we will focus on the FIPS technique and outline its progress from mechanism to applications. Compared with the nanosecond laser pulses, the femtosecond pulse that is focused on medium will induce a series of distinct ionization and phase transitions, due to the thermal conduction from the irradiated area, and plasma-laser interactions can be reasonably ignored. The physical scenario describing the interaction between femtosecond pulses and medium consists of various processes in different spatial and temporal scales, such as light absorption, non-linear ionization, plasma formation, electron-phonon collision, and ablation, where plasma and ablation dynamics are of great importance in FIPS. During the plasma that is induced by femtosecond laser, the fluorescence spectra that is emitted from the excited intermediate products such as atoms, ions, and molecules can be collected and applied to identify th material composition. The femtosecond laser-induced plasma in different medium is a complicated physical phenomenon, that is affected by the physical parameters of both laser pulses and matter. Here we just briefly summarize the major physical processes in the solid, liquid, and gas.

### 5.1. Femtosecond Laser-Induced Plasma in Solid

**Figure 15:**
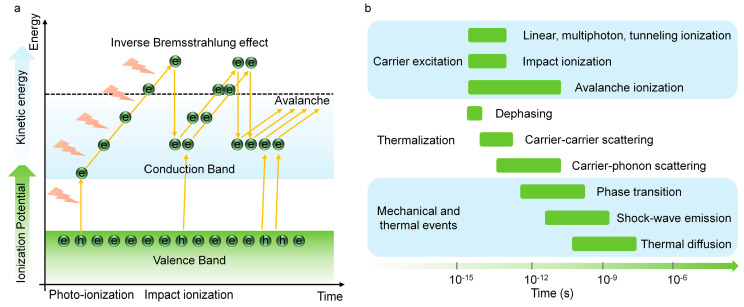
Femtosecond laser-induced plasma in solid. (**a**) Schematic diagram of photoionization, inverse bremsstrahlung effect, impact ionization, and avalanche ionization during the plasma generation. (**b**) Timescale of the physical phenomena that are associated with the interaction between femtosecond laser and solid.

When the intense femtosecond laser is incident on a solid, the ionization, i.e., carrier excitation occurs at the first stage. For a metal, photons energy of incident pulses can be absorbed by free electrons via the inverse bremsstrahlung process and generate non-equilibrium electron distribution that do not obey the Fermi–Dirac statistics after dephasing of the coherent electrons that are driven by a laser field. Whereas in the transparent material such as a semiconductor, the free electrons are excited from valence band to conduction band through linear or nonlinear multi-photons absorption (the involved photons are determined by bandgap and photon energy). In the case of high laser intensities, tunneling ionization (TI) also takes place in the superficial layer of a sample [227,228]. The governing photoionization mechanisms such as multiphoton ionization and tunneling can be judged by the Keldysh theory based on the field strengths and frequencies of the light field [229,230]. Under a sufficiently large light field, the generated free electrons can absorb photons through the non-resonant process of inverse bremsstrahlung. Then, after a sequence of inverse bremsstrahlung absorption events, the accumulated kinetic energy of the carrier is sufficiently large to produce another free electron through impact ionization. Providing the laser energy is high enough to continuously supply the losses of free electrons generation and impact events, then more and more free electrons continue to acquire photons energy through an inverse bremsstrahlung process and induce impact ionization and once again, leads to an avalanche growth of carrier similar to a chain reaction, that is, ‘avalanche ionization’ or ‘cascade ionization’. During this period, electrons absorb energy via an inverse bremsstrahlung effect, and avalanche ionization prevails over other ionization mechanisms. These ionization processes are briefly summarized in Figure 15a.

As depicted in Figure 15b, the electron subsystem will reach a thermal equilibrium with a high equivalent statistical temperature through electron–electron collisions in a few or tens of femtoseconds, while the peripheral regions of the lattice are still cold. Subsequently, electron-lattice thermalization occurs via phonon-electron interaction on a picosecond time scale. Generally, the electrons and phonons subsystem with different temperatures can be well described by the famous two-temperature model [231,232,233]. After thermalization, the heated lattice will be melted when the temperature reaches melting point. The melting depth can reach hundreds of nanometers depending on laser fluence and material [234,235]. Note that the direct evaporation to gas phase will occur in superficial layers (depth of ~10 nm) when the high temperature on the scale of 10^4^ K is produced. Therefore, the complicated phase transitions such as phase separation, gas–liquid mixing, metastable liquid, supercritical regime or atomization, and phase explosion will occur, depending on the laser pulse and matter [236,237,238,239]. In this stage, tensile stress that is caused by thermoelastic and shock waves leads to the formation and coalescence of voids, thereby giving rise to mechanical decomposition and spallation over tens of picoseconds [234,240]. Subsequently, a pressure or a shock wave separates from the dense, hot focal volume within the nanosecond scale, and the thermal energy diffuses out of the focal volume on the microsecond timescale [241,242,243]. Ultimately, the above phase transition processes at a sufficiently high energy will leave accurate and permanent structural changes on the materials, which has been extensively utilized to micromachining, especially for transparent, crisp, and hard material [244,245,246].

### 5.2. Femtosecond Laser-Induced Plasma in Liquid

The interaction between a femtosecond laser and water is the most fully studied among the various liquids, due to water being the most common solvent and the main component of biological tissues, which is of great importance on femtosecond laser nanosurgery of cells and tissues. Therefore, here we take water as an example to quantitatively describe the basic physical scenario of the liquid ionization that is induced by a femtosecond laser. To describe the ionization and evolution process in water, Sacchi has proposed that water should be treated as an amorphous semiconductor and the excitation energy ∆ regarded as the energy that is required for a transition from the molecular orbital into an excitation band (band gap 6.5 eV) [247,248,249]. Femtosecond laser-induced plasma in liquid water actually involves both ionization and dissociation of water molecules.

**Figure 16:**
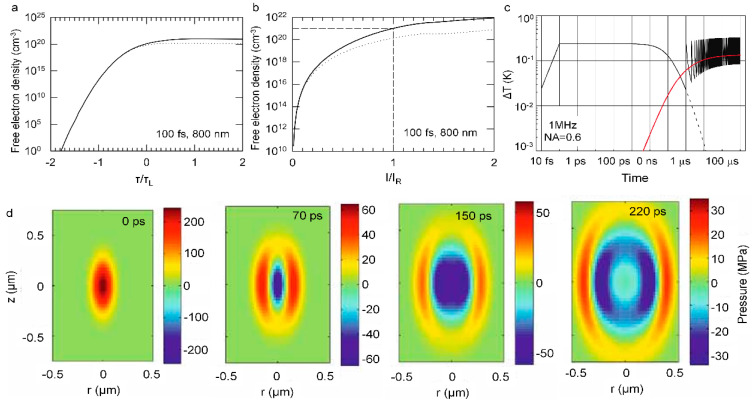
(**a**) Temporal evolution (normalized by pulse duration τ_L_) and (**b**) intensity dependence (the irradiance I is normalized by the threshold irradiance I_R_) of the free-electron density during the laser pulse at the optical breakdown threshold for 100 fs, 800 nm pulses. (**c**) Temperature evolution at the center of the laser focus that is produced by 1 MHz, 100 fs, 800 nm focused into water. (**d**) Thermo-elastic stress evolution for a peak temperature of 200 °C, slightly above the threshold for bubble formation [252].

Similar to the solid semiconductor, the free electrons can be produced by photoionization (multiphoton ionization or tunneling) and by impact ionization. Providing the electron density and the supplied laser energy is high enough, the cascade avalanche ionization will be initiated. Through the above photoionization, impact ionization, and cascade avalanche ionization processes, amounts of carriers, i.e., plasma are formed. Generally, the plasma continuum are often regarded as the breakdown criterion for nano- and picosecond pulses. Here, plasma emission for the shorter femtosecond laser pulses is not in the visible region. Therefore, other attendant phenomena such as shock-wave emission and cavitation bubble are often served as the breakdown signal in aqueous media [250,251,252]. Compared with the longer pulses, the femtosecond pulses will launch an extremely different plasma formation dynamic: the ultrastrong peak intensity of femtosecond pulse favors the generation of free electrons through multiphoton ionization and thereby the avalanche ionization can be initiated at a fairly large pulse intensity range below the breakdown threshold [252]. Such steadily increasing free electron density with laser intensity, provides an opportunity for precise control of plasma density and damage mechanism in liquid (Figure 16a,b).

Subsequently, the energy of electron subsystems transfer to the “cold” surroundings on the picosecond time scale, obeying the two-temperature model (Figure 16c). The stress wave that is induced by rising temperature that is emitted from a finite volume within the extended medium should contain both compressive and tensile components to ensure the momentum conservation. Once the temperature rises to a critical point (a temperature rise of 131.5 °C, well below the superheat and thermal damage threshold [252,253]), the thermo-elastic tensile stress leads to material fracture, i.e., cavitation bubble formation in aqueous media, illustrating the laser-induced effects becoming more disruptive and the breakdown occur (Figure 16d). Due to leading free electron generation, the plasma-mediated chemical or photochemical effects cannot be clearly separated from thermal, and thermo-mechanical effects. Thermo-elastic stresses that are caused by the temperature rise are confined in the focal volume, and due to the acoustic propagation distance is much smaller than the laser action region during this time interval of picoseconds.

### 5.3. Femtosecond Laser-Induced Plasma in Gas

In atmosphere, the femtosecond laser pulse propagates and converges into a small volume through external focus and self-focus, the gas molecules are ionized, dissociated, and recombined at a high intensity, producing substantial plasma to overcome the catastrophic collapsing of the laser beam. The dynamic balance of these effects ensures that the pulse can propagate in a form similar to a bullet or soliton through a long distance up to the kilometer range, dubbed “filamentation” (see Section 2 for more details). As supplementary, here we focus on femtosecond laser pulse-induced plasma in gas, especially the ionization and dissociation of gas molecules, which is the most critical process for gas-based FIPS.

Due to tunnel ionization of gas atoms or molecules being similar to multiphoton ionization in the sense that both are high intensity effects, multiphoton and tunnel effects dominate the ionization process under the ultrastrong light intensity, providing the photon energy is less or much less than the ionization potential or the work function. Therefore, the gases could be in the multiphoton or the tunnelling regime when using femtosecond laser pulses to ionize gases, depending on Keldysh theory and the combined condition of the laser and the ionization potential of gas [11]. As is well known [11], the density of particles (atoms, molecules) in gas (~3 × 10^19^ cm^−3^ in atmosphere) is about three orders lower than that in a condensed medium (~10^22^ cm^−3^ in solid and liquid), which can lead to a greater difference of breakdowns between gases and condensed matter. Most directly, the breakdown threshold of laser intensity in gas is much higher than that in solid and liquid. Additionally, the mean free time of free electrons from the multi-photon or tunnel ionization in atmosphere is about several hundred femtoseconds (but it is <1 fs in condensed matter), which is longer than the femtosecond pulse duration [11,254]. Generally, no inverse bremsstrahlung and cascade or avalanche ionization could take place in gases at one atmospheric pressure at the intensity of ~10^13^ W/cm^2^.

**Figure 17:**
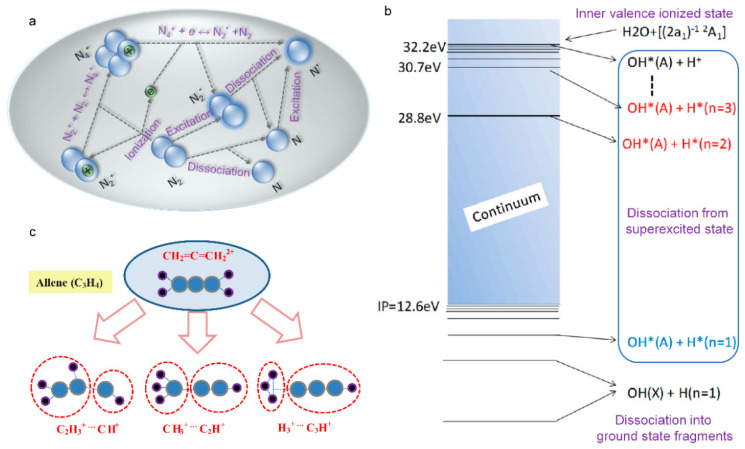
Schematic diagram of the dissociation of N_2_ (**a**), H_2_O (**b**) [255], and allene (**c**) [14] in atmosphere. IP: ionization potential.

Besides ionization, the inevitable dissociation of gas molecules inside the femtosecond laser filament has a decisive influence on the fluorescence emission. For the femtosecond laser filamentation in the atmosphere, as shown in Figure 17a, the main components of nitrogen molecules can be excited to N_2_* and ionized into electrons and N_2_^+^, then the N_2_^*^ can be further divided into N* and atomic N, as well as the other possible channel that is the N_2_ molecule is dissociated into atomic nitrogen (N) in ground state which is subsequently excited into N* by light field or by collision. The primary reactions, N_2_^+^ + N_2_ ↔ N_4_^+^ and N_4_^+^ + e ↔ N_2_* +N_2_ are responsible for populating the excited state of N_2_ (N_2_*) [255,256]. Meanwhile, the dissociation of O_2_ molecules can produce fragments of atomic O and excited O* [96]. In a moist atmosphere, the widespread hydrone molecules can be directly dissociated into fragments of OH and H radicals, then the NH radical can be formed by a chemical reaction of excited atomic nitrogen and H radical (Figure 17b). The fluorescence of OH and NH radicals are the important indicators to calibrate the air humidity [255,256]. Similarly, the various decomposition channels of air pollutants inside the filament were also demonstrated in previous reports. Figure 17c depicts the three critical decomposition channels of allene (C_3_H_4_) [14]. In a word, the molecular dissociation inside filaments determine the spectra of FIPS, in turn, the FIPS spectra can provide the direct experimental evidence for the microscopic dissociation dynamics.

## 6. Sensing Applications of Filament-Induced Plasma Spectroscopy

The feasibility of excitation and dissociation of molecules during filament-induced plasma for sensing applications has been extensively studied. In this part, we mainly summarize and discuss the sensing applications of FIPS from the following aspects including the analysis and monitoring of multi-gas pollutants, aerosol and micro-particles, multi-metallic alloy, geological and mineral matters, physicochemical reaction, and explosive and radioactive matters, etc.

### 6.1. Multigas Pollutants

**Figure 18:**
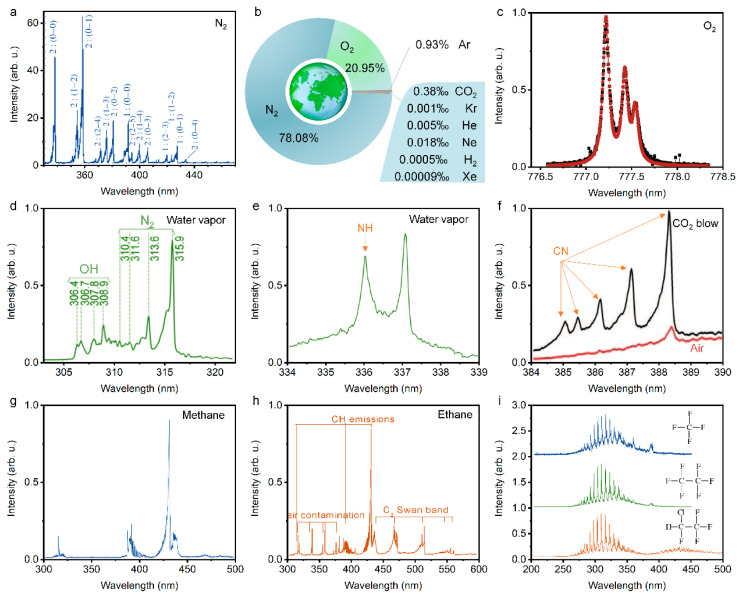
(**a**–**f**) Chemical composition (**b**) and corresponding FIPS spectra (**a**,**c**–**f**) in atmosphere [136,184,190,256,268]. (**g**–**i**) FIPS spectra of several representative organic pollutants in atmosphere [191,258,269].

Atmospheric environmental issues such as global warming, stratospheric ozone depletion, and photochemical smog formation is increasingly severe with the industrialization of mankind. Remote sensing of atmospheric species has attracted intensive interest, which can deepen our understanding of the complex mechanisms that lead to atmospheric or climatic changes, potential adverse health effects, and provide scientific guidance for comprehensively improving air quality. The natural atmosphere on the earth consists of three main components—nitrogen, oxygen, and argon, and the diverse trace gases including greenhouse gases carbon dioxide, methane, nitrous oxide, and ozone, and the noble gases such as neon, helium, krypton, and xenon (Figure 18b). Additionally, air can contain as much as 5% water vapor and droplets, more commonly ranging from 1–3%, depending on the regional climate. More importantly, human activity such as industrial production and daily life can produce plentiful pollutant emissions in the atmosphere, including chlorine and its compounds, fluorine and its compounds, elemental mercury vapor, sulfur dioxide, and hydrogen sulfide. By using filament-induced fluorescence technique, various atmospheric species such as hydrocarbon gases (CH_4_, C_2_H_6_ C_2_H_2_) (Figure 18g,h), carbon dioxide (Figure 18f), water vapor molecules (Figure 18d,e), and chemical agents (acetone), can be identified or even quantitatively measured [99,190,255,256,257,258,259]. The combination of this detection strategy with LIDAR could allow the long-range monitoring of several atmospheric species with a single laser source, eventually leading to a better understanding of chemical and dynamic processes affecting global warming, ozone loss, tropospheric pollution, and weather prediction.

During femtosecond laser filamentation in atmosphere, the main components of N_2_ and O_2_ molecules can be excited and ionized and give rise to serial typical emissions (Figure 18a,c), which are mainly assigned to three spectral band systems: the second positive band system of N_2_ (C^3^Π_u_-B^3^Π_g_ transition), the first negative band system of N_2_^+^ (B^2^Σ_u_^+^-X^2^Σ_g_^+^ transition), and the characteristic Stark broadened atomic oxygen triplet that is centered at 777.4 nm (Figure 18c), where the emission lines of the first negative band and the second positive band are simply labelled as 1 and 2 [99,136,184,260,261,262]. The clean fluorescence emission-free of the plasma continuum that is induced by femtosecond laser filament facilitate the high sensitive multicomponent analysis by FIPS. Subsequently, considering the different atomic emissions of C, H, O, and N [263], the simultaneous detection and identification of multiple mixed gases with air at atmospheric pressure were performed.

Carbon dioxide (CO_2_) and hydrocarbon gases (such as CH_4_) are the key component greenhouse gas that lead to global warming, and are major urban pollutants that are emitted from motor-vehicle exhaust. As shown in Figure 18f, the intense filaments can induce the dissociation of N_2_ and CO_2_ molecules and then recombine with each other to create CN radicals, populating the excited state and emitting a series of fluorescence [190]. Therefore, the CO_2_ molecules in the atmosphere can be tracked by the intensity of CN radicals. Although methane (CH_4_) is a colorless, odorless, non-toxic gas, its concentration can reach up to 5 × 10^4^ ppm (5%) in a facility such as a poorly ventilated deep mine shaft, which can easily be ignited and result in a fiery explosion by a spark. The high intensity femtosecond laser filaments can dissociate the CH_4_ molecules into small fragments which emit characteristic fluorescence. Backward CH radical fluorescence can be used to quantitatively analyze the pollutant concentration. It was estimated that the detection limit of 3σ will be reached at about 0.85 km for the filament length of L = 20 m. This concentration of 5% corresponds to the practical limit at which it will become potentially explosive (5% to 15%, for methane in air) [257]. Additionally, the hydrocarbon gases with more complex molecular structures such as toluene molecules (C_7_H_8_), can be also fragmentated and ionized into various hydrocarbon ions C_m_H_n_^+^, with serious CH radical emission. The yields of ionization and fragmentation can be enhanced by linear (focusing geometry) and nonlinear (small-scale self-focusing and self-phase modulation) effects during the filamentation [259,264]. For another kind of alcohol molecule such as methanol, ethanol, phenylcarbinol, and glycol, besides the bands of 420–450 nm and 385–405 nm come from the A^2^Δ, B^2^Σ, and C^2^Σ-X^2^Π transitions of the CH radical, the spectral lines in the region of 436–644 nm corresponding to C_2_ Swan band can be also clearly observed, illustrating the C_2_ radical was produced during fragmentation [258]. Clearly, such ubiquitous fragmentation complex molecules make it challenging to selectively identify the molecules, but also provide an effective method to quantitatively detect a class of compounds. In spite of this, the FIPS combined with genetic algorithm had been presented for multicomponent atmospheric sensing [258]. Specifically, the spectral signatures and the signal strengths of two molecules such as CH_4_ and C_2_H_2_ at different concentrations were individually collected and stored in the genetic algorithm database. Then, the arbitrary mixture of these two gases were analyzed within a precision of 25% by using a genetic algorithm.

Due to their central role in ozone layer depletion and global warming, halogenated alkane (CFCs, chlorofluorocarbons; halons; HCFCs, hydrogen containing chlorofluorocarbons; HFCs, hydrofluorocarbons; PFCs, perfluorinated compounds) pollutants are of particular interest, which has been monitored by various sensitive and selective analysis techniques off-line and on-line. It was demonstrated that characteristic difluorocarbene radical (CF_2_) fluorescence in the UV vis can be generated inside a femtosecond laser-induced filament for different halocarbons (Figure 18i). The different fluorescence lifetimes provide an opportunity to temporally resolve the characteristic fluorescence of CF_2_-containing halocarbons from that of background species such as N_2_, therefore enhancing the signal-to-noise ratio. With the current experimental setup, the 3σ detection limit of 500 ppmv for C_2_F_6_ in air was calculated, which is limited by the remaining background N_2_ emission. An instrumental detection limit of 8 ppmv has been calculated with the current calibration sensitivity [191].

### 6.2. Aerosol and Micro-Particles

There are tens of millions of solid particles and liquid droplets that will be inhaled into our body during every breath, even for the clear air [265]. These ubiquitous micro-specks of matter in our surroundings are known as aerosols, suspended and drifting in the atmosphere from the stratosphere to the earth surface, ranging from a few nanometers to several tens of micrometers [266]. It was dubbed as particulate matter PM 2.5 or PM 10 by meteorologists and government depending on the size. Generally, the aerosols can be classified as sulfates, organic carbon, black carbon, nitrates, mineral dust, and sea salt, but complex mixtures are more common due to the clumping of different aerosols [265]. Despite anthropogenic aerosols being only ten percent, the hazardous ingredients such as sulfates, nitrates, black carbon, and other particles from automobiles, incinerators, smelters, and power plants in fact have a great impacts on our climate and our health [267]. In the past decades, severe haze events with exceedingly high levels of fine aerosols occur frequently over in the North China Plain (NCP), exerting profound impacts on human health, weather, and climate. The identification and quantification of the sources, formation, and transformation of the aerosol can provide an insight into the haze formation mechanisms, which is critical to formulate effective mitigation policies.

The hygroscopicity of aerosols does not only reflect the variations in the particle chemical composition but also regulates the aqueous chemistry [270,271,272]. Therefore, water vapor content, that is humidity, is particularly a important parameter for haze aerosol evolution. The monitoring of atmosphere humidity can be performed by FIPS through fluorescence detection of OH and NH radicals, where the former are the directly dissociated fragments of water vapor molecules (see Ref. [255] for detailed physical picture) and the later can be ascribed to the recombination process of the dissociated N_2_ and water vapor molecules [256]. Clearly, the water vapor molecules were the essential physical and chemical reactants of OH and NH radicals inside the filament, therefore the water vapor concentration can be calibrated through filament-induced fluorescence intensity of these radicals [268].

Actually, complex mixers such as sulfate, nitrate, and metallic ion often dissolve in water vapor and form various aqueous aerosols. The FIPS is a sensitive sensing technique to remotely retrieve the composition of microdroplets in clouds that are located at a distance. The pioneer experiment with sodium demonstrated that it is possible to detect and identify, at a distance of several tens of meters, a single component that is dissolved at low concentration in a cloud of aerosol droplets (ppm-level sodium can efficiently be excited and observed 70 m away from the detection system) [192,273]. Subsequently, a cloud of aqueous aerosols containing a mixture of PbCl_2_, CuCl_2_, FeCl_2_, and NaCl was probed by remote FIPS [274], where the first three items are mainly of interest to the mining industry, especially PbCl_2_ are toxic materials and constant respiratory exposure can lead to many related diseases [275]. The spectrally narrow atomic transitions that are excited by the low-density plasma can be observed clearly, illustrating that FIPS can be used to simultaneously recognize and distinguish every single metallic constituent that is dissolved inside such a cloud [274,276]. Recently, the aqueous aerosol droplets containing various metallic ions with diameters of 0.8–2.0 μm were systematically detected by filamentation of femtosecond laser pulses (60 fs, 800 nm, 4.4 mJ) in weak focusing mode by a lens with a focal length of 500 mm. Similarly, soil and water contamination with heavy metals by human activities such as mining, fossil fuel consumption, fertilizer and pesticides, electronic waste, and industries including metallurgy, textiles, and refineries, etc. is also a severe problem, which can be also detected by FIPS [277]. As shown in Table 1, the limits of detection (LOD) for different metallic ion were summarized [192,277,278,279,280,281,282,283,284,285,286,287,288,289,290,291,292,293,294,295,296,297,298].

Apart from the gas and liquid molecules, solid particles such as smog and smoke are vital components of aerosols, connected with the photo-chemical smog and current hazy weather. By using the FIPS technique, the fluorescence emission from a distant cloud of smoke had been investigated [299]. The smoke, produced from burning mosquito coils, located at a distance of 25 m from the laser source and LIDAR detector can be identified by the emission of CN, CH, and C_2_ molecular fragments. Similarly, some very similar agricultural activity-related micro particles, namely barley, corn, and wheat grain dusts, can be also remotely detected and distinguished by the FIPS [300]. All the species showed identical spectra, namely those from molecular C_2_ and CN bands, as well as atomic Si, C, Mg, Al, Na, Ca, Mn, Fe, Sr, and K lines, revealing similar chemical compositions. However, the different element abundances lead to intensity ratio variations among the elemental lines, which was used to distinguish these three biotargets with a good reproducibility.

**Figure 19:**
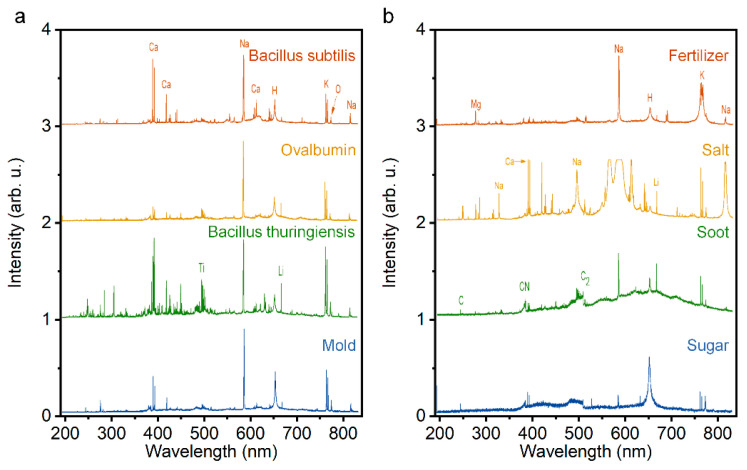
LIBS spectra of biological warfare agent surrogates (**a**) and non-biological interferents (**b**), including fertilizer, iodized salt, diesel particulate (soot), and granulated sugar [302].

In addition to the pressing need for atmospheric environmental monitoring, the recent use of sarin gas in the 1995 Tokyo subway attack and the distribution of anthrax spores through the U.S. Postal Service in 2001 demonstrate the urgency of chemical and biological warfare agent sensing [301]. Comparing common immunological and nucleic acid-based detection methods, FIPS can provide a remote, real-time detection of biological warfare agents in aerosol (potential method for disseminating biological agents) or other forms. In addition to defense applications, the fast and accurate detection and classification of biological aerosols could be important in preventing the spread of disease in hospitals and for monitoring outdoor allergy-causing molds and pollens. Considering the different ratios of atomic spectral lines (Figure 19a,b), biological agent simulants can be discriminated from natural background aerosols by principal components analysis [302]. As yet, the standoff detection of similar biological materials such as egg white, yeast powder, grain dusts, and animal tissues were generally limited in solid targets, and the biological aerosol analysis by FIPS deserves further study [300,303,304].

### 6.3. Multi-Metallic Alloy

As is well known [305], the shortening of laser pulse durations depositing on the solid materials can produce a reduced heat-zone with little thermal diffusion, avoiding uncontrollable and often undesirable material modification and removal. Therefore, a lower threshold and larger efficiency of material ablation can be obtained with high precision and minimal damage. In the past two decades, the ablation and accompanied fluorescence of high purity metal and multi-metallic alloy by femtosecond laser or filaments have been extensively studied, owing to its critical role on the promising accurate femtosecond laser micro-nano machining. Meanwhile, the metallic atomics spectral emission with a high efficiency and narrow linewidth that is induced by femtosecond laser filament have attracted intensive attention, and the filament-induced fluorescence was developed as an high sensitive remote detection method to analyze the constituents of multi-metallic alloys. 

In 2001, the pioneer study of the time-resolved LIBS of plasmas that were produced by ultrashort laser pulses at atmospheric pressure, on aluminum alloy targets was reported [305]. The temporal behavior of specific ion and neutral emission lines of Al, Mg, and Fe has been characterized. The results show a faster decay of continuum and spectral lines, and a shorter plasma lifetime than in the case of longer laser pulses. The limits of detection are in the parts per million ppm range and are element-dependent. Subsequently, the advantages of remotely sensing metallic targets using FIPS was further discussed focusing on the temperature and electron density of the plasma that was ejected from a lead target in ambient air [193]. It was found that the electron density of 8 × 10^17^ cm^−3^ and the plasma temperature of 6794 K were obtained for a 20 ns time delay. This plasma temperature is two times smaller than that which was obtained in a typical ns-LIBS experiment (~13,000 K, averaged without any temporal resolution), using an ArF excimer laser at 193 nm, indicating a low background blackbody continuum and a high signal-to-noise ratio of emission spectral line.

In order to get more insight into the application of the FIPS technique, the estimation of ultimate detection distance that was based on the data was performed in many experiments [306]. Due to the intensity clamping inside the filament, the local FIPS signal can be approximated as distance-free. The influence of distance R on the amount of the signal S that was collected by the LIDAR system will obey the rule: S ∝ 1/R^2^. Then, the intensity of the atomic spectral line versus the distance can be estimated and plotted. Adapting the criterion of 3σ to define the detection limit, the kilometer detection range can be obtained generally. Here, σ is the standard deviation of the background spectrum. Note that the advanced data analysis technique such as the principal component analysis (PCA) and machine learning can further improve the performance of FIPS on the detection range, precision, and sensitivity [307].

### 6.4. Geological and Mineral Matters

Classifying geological and mineral substances can provide an insight into the temporal evolution of heterogeneous crustal geophysics, associated mineral endowments, and their genesis [308]. In addition, soil and rocks is an inevitable background for detecting nuclear and hazardous chemicals and explosive traces in real scenario. Therefore, the geological and mineral analysis is of great significance, and various methods such as wet chemical process, X-ray fluorescence (XRF), and inductively coupled plasma mass spectrometry (ICP-MS) were developed to quantify chemical parameters, weight percentage (wt%), and trace element concentration (ppm, ppb, and ppt levels) of rock suites, i.e., ultramafic, mafic, and felsic [309]. The occupational disease can be caused by mineral analysis in the field due to the direct or long-term exposure to hazardous ingredients. Here, the LIBS or FIPS can provide real-time, robust analysis of geological materials with a reduced operator’s risk [310]. In past decades, a wide variety of geomaterials, including garnet samples that were collected worldwide [311], obsidian samples from the southwestern United States [312], and a survey of carbonates, fluorites, silicate rocks, and soils [313], were analyzed and classified by LIBS or FIPS conveniently and accurately, combined with various classification algorithms such as soft independent modeling of class analogy (SIMCA), partial least squares (PLS), and lasso-regression [314,315,316].

**Figure 20:**
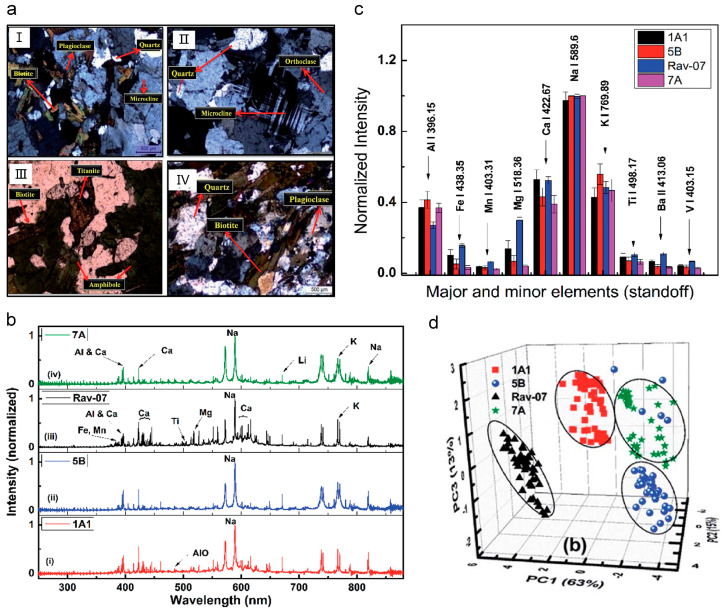
(**a**) Photomicrographs of (I) Tonalite-1A1, (II) Granite-5B, (III) Maficenclave-Rav-07, and (IV) Granodiorite-7A depicting the textures and modal mineral assemblages of different granitoids. (**b**) Typical normalized standoff FIPS spectra of granitoids in (**a**). (**c**) Atomic peak intensities of major elements for the granitoids in (**a**). (**d**) PCA score plots of the normalized FIPS spectra of granitoids in (**a**) [309].

In the earlier geological and mineral analysis, the portable and hand-held LIBS devices were commonly adopted. Recently, the advantages that are mentioned above of FIPS was also demonstrated in standoff investigations of geological samples [309]. In Ref. [309], four granitoids samples including tonalite, granite, mafic enclave, and granodiorite, that were collected from the Dharwar craton, Karnataka, South India, were analyzed and discriminated by the FIPS in two configurations: near-field by using tightly focused fs laser pulses and standoff by using loosely focused fs filaments. Figure 20a shows the photomicrographs of four granitoids samples, depicting the textures and modal mineral assemblages of different granitoids that were chosen for the standoff FIPS. According to the modal proportions of quartz, alkalifeldspar, plagioclase, and feldspathoid from the International Union for Geological Sciences (IUGS), the four samples were classified as Tonalite-1A1, Granite-5B, Mafic enclave-Rav-07, and Granodiorite-7A. Figure 20b illustrates the typical normalized FIPS spectra of four such granitoids samples, in the spectral regions 240–880 nm, respectively. Based on the standard NIST atomic database, the atomic emission spectra of the major and trace elements were identified and labelled. For clear and quickly comparison, the spectral intensities of the major (Si, Al, Fe, Mg, Na, Ca, and K), minor (Mn and Ti) and trace (Ba and V) elements were normalized by Na I 589.6 nm atomic emission and sketched in Figure 20c. Finally, the multivariate analysis method, PCA, was utilized to discriminate granitoids based on the FIPS spectra. The principal components (PCs) of the principal features for the classification were obtained, where the first three PCs account for 91% of the total variance (63, 15, and 13%) that was present in the dataset; that is, the normalized FIPS spectra of four granitoids samples in the 370–820 nm spectral range (Figure 20d). These results demonstrated a high accuracy classification of geological substances and minerals by FIPS, which will be further improved by adopting others supervised multivariate analysis algorithms (deep learning, PLS, SIMCA, etc.) and the spectra corrections (base-line corrections or continuum corrections) [317]. 

### 6.5. Physicochemical Reaction

Physical and chemical reaction sensing can provide an intuitive and visual evidence for the intermediate and final products and deepen our understanding of different kinds of reactions in vapor-liquid-solid phases. In the chemical industry and extreme physics (such as high temperature and pressure), there are numerous detrimental and hazardous physicochemical reactions that need to be monitored and optimized over the course of production and the product itself. In this respect, the FIPS is an effective technique to monitor various physicochemical reactions.

Returning to the filamentation itself, the filament with an ultrastrong clamped intensity of light, extreme density, and temperature of plasma can induce abundant physical and chemical reactions in various mediums. In sub-saturated atmospheres, the generation of three major gases, namely O_3_, NO, and NO_2_ during filamentation was demonstrated. These plentifully produced trace gases can initiate efficient oxidative chemistry of nitrogen, resulting in concentrations of HNO_3_ in the multipart per million range, which provides an insight into the efficient nucleation that is observed in laser filaments [318]. Similar to the dissociation and recombination of N_2_, O_2_, and CO_2_ molecules during filamentation in atmospheres, it can be expected that these complex species can also be excited and identified by FIPS [319].

**Figure 21:**
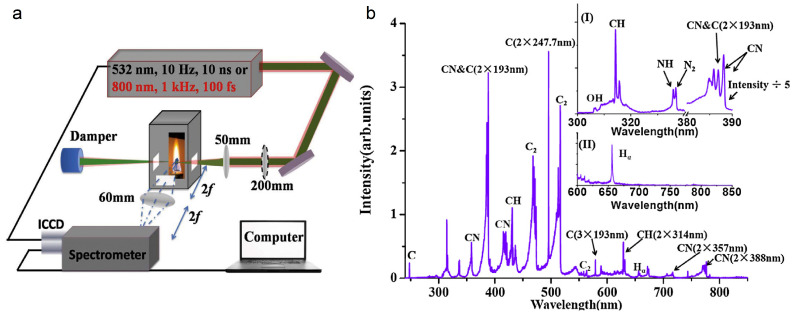
(**a**) Experimental setup and (**b**) typical FIPS spectrum ranging from 240 to 850 nm of the ethanol-air flame in atmosphere [327]. Insets: (I) zoomed-in spectrum in the range of 300–345 nm, and (II) spectrum obtained with a high-pass filter.

In combustion engineering, mapping the concentration distributions of intermediate species in combustion is essential to rationalize the physical and chemical nature of combustion systems for efficient combustion with low-pollution products. Although a large variety of intermediate species in combustion, such as OH, C_2_, CN, CH, and CO have been demonstrated by laser-based techniques, such as laser-induced fluorescence, coherent anti-Stokes Raman scattering, and polarization spectroscopy, most of these methods have been limited to sensing one species at a time [320,321]. It is worth mentioning that the simultaneous detection and identification of multiple intermediate species in a flame by FIPS was demonstrated in past decades [322,323,324,325,326,327,328,329]. Figure 21a,b show the experimental setup and the typical FIPS spectrum ranging from 240 to 850 nm of the ethanol-air flame in atmosphere [327]. Clearly, the species in the alcohol burner flame are very rich, consisting of not only atoms but also molecules. The three spectral bands at around 563, 516, and 416 nm are assigned to the Swan band (d^3^Π_g_−a^3^Π_u_) of the C_2_ radical, and that at around 408 nm belongs to the Deslandres-D’azambuja band of the C_2_ radical; the two spectral bands around 430 and 314 nm result from the A^2^Δ−X^2^Π and C^2^Σ−X^2^Π transitions of the CH radical, respectively; the spectral bands around 388 and 358 nm originate from the B^2^Σ^+^−X^2^Σ^+^ transition of the cyano radical, CN. In addition, several weak molecule band emissions such as N_2_ (337 nm), NH (336 nm), and OH (307 nm), as well as atomic lines of C(I) (247.7 nm) and Hα (656 nm) can be observed. Note that second- and third-order diffractions of the grating (such as double or triple C(I) line and CH bands) should be excluded. These findings illustrate that the FIPS technique is a powerful tool for the continuous monitoring of various physicochemical reactions.

### 6.6. Explosive and Radioactive Matters

Identifying and destroying the explosive devices and special nuclear materials from terrorists in advance is of top priority for many nations to safeguard their citizens [330]. Generally, the existing sensitive lab-based explosive trace detection techniques (ion mobility, mass spectrometry, etc.) is not in situ and is time-consuming [331,332]. The remote or standoff, highly sensitive, and real-time features have endowed FIPS with a great potential for detecting hazardous articles such as explosive and radioactive matters, which would otherwise be a great detriment to the national defense security and personal safety [333,334,335].

**Figure 22:**
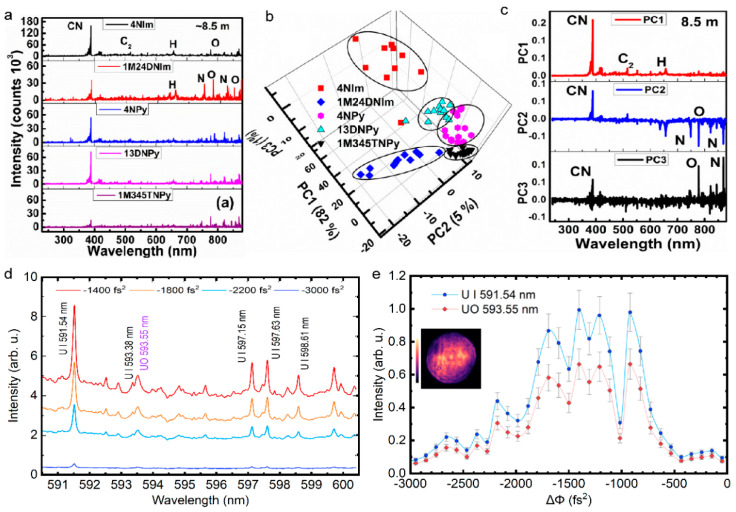
(**a**) Typical FIPS spectra of explosive molecules (nitroimidazoles and nitropyrazoles) that were obtained at 8.5 m away; (**b**) PC scores plot and (**c**) first three PCs for the processed LIBS spectra of explosives (nitroimidazoles and nitropyrazoles) that were obtained at 8.5 m [330]. (**d**) Typical uranium emission spectra for various GDD values of the incident laser pulse; (**e**) intensity of atomic (U I 591.54 nm) line and molecular (UO 593.55 nm) spectral band for various applied quadratic phases to incident laser pulse [345].

Figure 22a depicts the typical remote LIBS spectra ranging from 220 nm to 870 nm of different types of explosive molecules such as 4-nitroimidazole (4-NIm, C_3_H_3_N_3_O_2_), 1-methyl-2,4-dinitroimidazole (1M-2,4-DNIm, C_4_H_4_N_4_O), 4-Nitropyrazole (4-NPY, C_3_H_3_N_3_O_2_), 1,3-dinitropyrazole (1,3-DNPY, 1,3-DNPY), and 1-Methyl-3,4,5-Trinitropyrazole (1M-3,4,5-TNPY, C_4_H_3_N_5_O_6_) at a remote distance of ~8.5 m. All the important peaks can be identified and attributed to CN violet (B^2^Σ^+^−X^2^Σ^+^) and C_2_ Swan band: Δν = 0 band was clearly observed compared Δν = −1 in CN violet band, while Δν = 0 band (516.53 nm) was visible in C_2_ Swan band (d^3^Π_g_/a^3^Π_u_). To discriminate and classify the explosives from the FIPS spectra, a PCA was employed. The PCs score plot of the FIPS spectra and corresponding PCs are depicted in Figure 22b,c. Note that the first three principal components together account for 88% of the total variance (82, 5, and 1%) that is present in the experimental results. The major spectral features in classification are from PC1 (CN, C_2_, and H).

Additionally, recent studies indicate that the FIPS can be also applied to standoff isotopic analysis [336,337], which is of great interest in various fields including nuclear forensics and nonproliferation monitoring, archaeology, geology, biology, medical, and environmental applications. Specifically, the diet and movement of a population can be analyzed based on carbon, strontium, and lead isotopes in archaeology [338,339]; several isotopes can be employed to determine the location of origin and source processes of rocks, ores, soils, clay, and ceramics, etc. in geochronology [340,341]; and gunshot residue has been isotopically analyzed to distinguish different gun powders and types of ammunition in forensic science [342]. For the FIPS, the isotope composition of the sample under ambient atmospheric conditions can be extracted remotely by using the molecular radiation transition that is formed by the association of atoms (ions) in the sample vaporization or laser ablation plume [343]. Focusing on radioactive releases in nuclear systems, remote or standoff sensitive and the rapid detection of radioactive matters is conducive to maintain the security of nuclear power plants, enrichment, and reprocessing facilities, and especially to detect undeclared enrichment activities that violate global nonproliferation treaties [344]. Uranyl fluoride (UO_2_F_2_) is a compound which forms in the reaction between water and uranium hexafluoride. In 2018, FIPS was performed to detect UO_2_F_2_ in standoff mode, which has a potential application in guiding remediation efforts around enrichment facilities. Subsequently, the remote detection of uranium was demonstrated. Meanwhile, the initial location of filamentation can be controlled by applying a negative pre-chirp to optimize the emission signal from the uranium [345]. Figure 22d depicts the typical uranium emission spectra for various group delay dispersion (GDD) values of the incident pulse. Figure 22e presents the spectral emission of atomic (U I 591.54 nm) and molecular (UO 593.55 nm) uranium as a function of the initial chirp.

Besides the great applications that are mentioned above, it was demonstrated that FIPS also has a promising prospect in many other areas such as nondestructive analysis of cultural heritage [346], identification of composite graphite materials [347], and the discrimination of willow, pine, and poplar trees and their growing environments [348]. With opening our imagination, there are numerous practical application scenarios we could explore for such a versatile tool, such as remotely identifying toxic or dangerous airborne materials from the smog that is generated by enemy explosions in the military, monitoring the presence of fires or suspicious clouds and prevent large scale forests devastations in wooded areas, and verifying that polluting companies respect their agreements by mobile FIPS systems (truck, helicopter, or satellite) [299].

## 7. Conclusions and Perspective

Toward remote sensing in complicated conditions, we have mainly reviewed recent research advances in sensing with femtosecond laser filamentation from basic physics and manipulated methods of filamentation to the representative filament-based sensing techniques and application scenarios. Although femtosecond laser filamentation encompasses abundant nonlinear optical phenomena, including diffraction, dispersion, Kerr self-focusing, plasma defocusing, supercontinuum generation, super clean fluorescence emission and amplification, and self-pulse compression, etc., it can be briefly considered as the dynamic balance between the Kerr self-focusing and self-generated plasma defocusing. Owing to exotic properties such as intensity clamping, mode self-cleaning, pulse self-compression, self-healing, long-range filamentation and the generated ultrabroad spectra ranging from ultraviolet to terahertz bands, the femtosecond laser filamentation provides new opportunities for remote sensing. Additionally, the various spatio-temporal shaping techniques for femtosecond laser pulses can adjust and control the location, length, and plasma density of the filament, facilitating that the filamentation-based remote sensing is performed according to our requirements. Therefore, in the past two decades, versatile sensing technologies that are based on filamentation including FIPS, filamentation-based white-light LIDAR, filamentation-assisted terahertz remote sensing, and filament-driven impulsive Raman spectroscopy have been proposed, where the FIPS has attracted more intensive interest. The physical mechanism of femtosecond laser-induced plasma in transparent optical media (gases, liquids, and solids), and the various applications including the analysis and monitoring of multi-gas pollutants, aerosols and micro-particles, multi-metallic alloy, geological and mineral matters, physicochemical reaction, explosive and radioactive matters, etc., are investigated extensively.

Nowadays, there still remains opportunities and challenges from principle to application toward the filamentation-based remote sensing. Firstly, although the filamentation at the distance of kilometers in atmosphere has been demonstrated, the remote sensing and imaging of multicomponent pollutants and targets still remains challenging owing to the inevitable turbulence and ultralow concentration. Moreover, the extremely long filament during remote filamentation greatly limits the spatial resolution along the propagation direction for sensing. Therefore, the intensive and shorter filament would be expected for remote sensing. Secondly, the development of novel physics and technologies provide more opportunities to overcome the bottleneck of filamentation-based remote sensing. For example, the deep learning-assisted spectra analysis can significantly enhance sensitivity, and the feedback-controlled spatio-temporal shaping system of laser pulses can optimize the filament quality by combining artificial intelligence and a spatial light modulator. Additionally, the feasibility of the remote filamentation from an earth-orbiting satellite has been demonstrated theoretically [349], therefore, the new remote sensing tool for atmospheric, earth-orbiting white-light LIDAR, is more attractive, which enables the global monitoring of various atmospheric constituents including trace gases and aerosols at various atmospheric depths from the femtosecond laser source that is equipped on a spacecraft platform.

## Figures and Tables

**Table 1:** Limits of detection by FIPS of metallic ion in aerosol droplets or other conditions.

ELEMENT	λ (NM)	LOD (MG/L)	COMMENTS
**Cd**	228.802	0.029 [277]	Electrochemistry enrichment
	228.802	0.0031 [279]	Electrochemistry enrichment
	226.502	4.43 [280]	Water
	508.58	0.420 [281]	Chemical replacement
	226.50	0.4 [282]	Aqueous solution
	226.50	7 [283]	Engine oil in paper substrate
	226.50	129 [284]	Ca(OH)_2_ substrate
	226.50	0.59 [285]	Wood slice substrate
	226.50	10 [283]	Liquid jets
**Cr**	425.43	0.087 [277]	Hydrogel-based solidification
	425.43	0.004444 [286]	Hydrogel-based solidification
	425.43	1.26 [287]	Liquid jets
	520.84	0.025 [281]	Chemical replacement
	425.43	0.00129 [288]	Membrane separation
	360.53	0.52 [282]	Aqueous solution
	360.53	29 [283]	Engine oil in paper substrate
	360.53	0.018 [289]	Filter paper substrate in inert gas
	360.53	1.2 [284]	Ca(OH)_2_ substrate
	360.53	0.034 [285]	Wood slice substrate
	360.53	10 [290]	Liquid surface
	360.53	43 [283]	Liquid jets
**Cu**	324.75	0.012 [277]	Enrichment with aluminum target
	324.75	0.016 [291]	Enrichment with aluminum target
		0.131 [292]	Enrichment with wood
		0.25 [281]	Chemical replacement
		0.00259 [288]	Membrane separation
	324.70	0.39 [282]	Aqueous solution
	324.70	4 [283]	Engine oil in paper substrate
	324.70	0.01 [293]	Carbon substrate in inert gas
	324.70	0.029 [285]	Wood slice substrate
	324.70	7 [290]	Liquid surface
	324.70	2.4 [283]	Liquid jets
**Ni**	341.476	0.083 [277]	Electrochemistry enrichment
	341.476	0.0017 [294]	Electrochemistry enrichment
		0.28 [295]	Enrichment with graphite planchets
**Pb**	405.78	0.125 [277]	Electrochemistry enrichment
		2.93 [296]	Ultrasonic atomizer
		0.118 [281]	Chemical replacement
	405.78	1.27 [282]	Aqueous solution
	405.78	18 [283]	Engine oil in paper substrate
	405.78	0.075 [289]	Filter paper substrate in inert gas
	405.78	20 [284]	Ca(OH)_2_ substrate
	405.78	10 [293]	Carbon substrate in inert gas
	405.78	0.074 [285]	Wood slice substrate
	405.78	100 [290]	Liquid surface
**Zn**	206.20	0.049 [277]	Water
	206.20	13.67 [286]	Water
	202.55	0.51 [282]	Aqueous solution
	202.55	5 [283]	Engine oil in paper substrate
	202.55	21 [284]	Ca(OH)_2_ substrate
	202.55	1 [293]	Carbon substrate in inert gas
	202.55	120 [290]	Liquid surface
	202.55	11 [283]	Liquid jets
**Mn**	257.61	0.13 [282]	Aqueous solution
	257.61	4 [283]	Engine oil in paper substrate
	257.61	0.036 [285]	Metallic surface
	257.61	6 [297]	Liquid surface
	257.61	6 [283]	Liquid jets
	403.08	32.2 [278]	Aqueous aerosol
**Al**	396.15	12.1 [278]	Aqueous aerosol
	396.15	0.19 [298]	Aqueous aerosol
**Ba**	553.35	41.7 [278]	Aqueous aerosol
	553.35	0.08 [298]	Aqueous aerosol
**Ca**	422.67	10.0 [278]	Aqueous aerosol
	422.67	0.01 [298]	Aqueous aerosol
**Mg**	285.21	7.3 [278]	Aqueous aerosol
	285.21	1.0 [298]	Aqueous aerosol
**Na**	588.99	0.7 [278]	Aqueous aerosol
	588.99	3.0 [192]	Aqueous aerosol
	588.99	0.0009 [298]	Aqueous aerosol

## Data Availability

The data presented in this study are available on request from the corresponding author.
